# Spiking Neurons Derived from Proteinoid and Bacteriorhodopsin

**DOI:** 10.1021/acsabm.5c00964

**Published:** 2025-08-18

**Authors:** Panagiotis Mougkogiannis, Andrew Adamatzky

**Affiliations:** Unconventional Computing Laboratory, 1981 University of the West of England, Bristol BS16 1QY, U.K.

**Keywords:** Bacteriorhodopsin, Proteinoids, Visible Light, Spike Computation, Photosensitivity, Neuromorphic

## Abstract

We investigate proteinoid-bacteriorhodopsin complexes as biomolecular spiking neurons for neuromorphic computing applications. Our system shows strong photoresponsive behavior through the integration of bacteriorhodopsin with self-assembled proteinoid structures. Measurements show that proteinoid-bacteriorhodopsin complexes have greater electrical activity (10.77 ± 2.21 mV) than proteinoids alone (4.34 ± 4.47 mV). The complexes have wavelength-dependent responses to 5 Hz optical stimulation. Green light (λ ≈ 520 nm) produced the strongest amplitude (7.31 ± 1.49 mV). Temporal analysis shows a consistent periodicity (τ ≈ 645 s) across wavelengths. This indicates stable oscillatory mechanisms. Random walk computations show distinct spatiotemporal patterns, suggesting potential applications in light-controlled molecular computing. These findings demonstrate that proteinoid-bacteriorhodopsin complexes are promising candidates for bioinspired computing and offer possibilities for developing biomolecular information systems.

## Graphical abstract

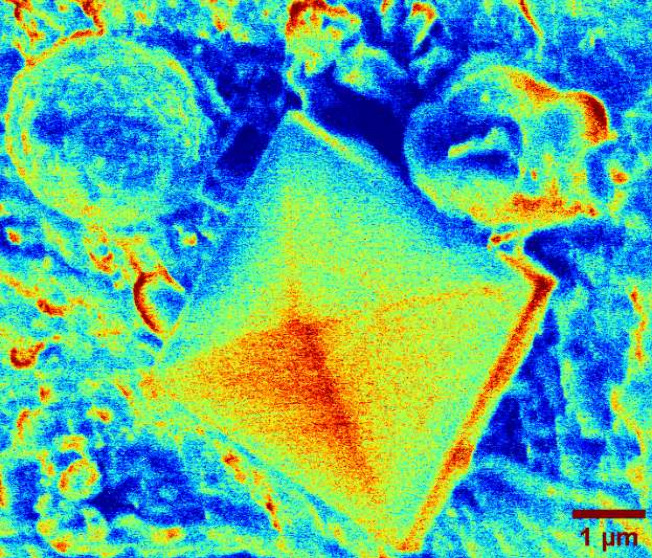

## Introduction

Bacteriorhodopsin^1–3^ is a light-driven proton pump found in *Halobacterium salinarum*. It is a key model for studying light-activated protein dynamics.^4^ Its structure has been extensively studied using X-ray crystallography.^5^ Figure S1 shows the monomeric structure of bacteriorhodopsin in its native membrane. The retinal chromophore and key amino acids in the proton-pumping mechanism are visible (Figure S1). This integral membrane protein comprises a protein component covalently linked to a retinal chromophore. Light absorption triggers photoisomerization of retinal. This starts a series of changes that drive proton transport across the membrane.^6^ Bacteriorhodopsin’s structure consists of seven transmembrane α-helices with well-defined proton translocation pathway. This structure allows efficient, light-driven proton pumping.^1,7–9^ So, it is a great candidate for bioelectronic uses. Upon photoactivation, bacteriorhodopsin undergoes a photocycle with distinct intermediates (K, L, M, N, and O states).^10,11^ Each has unique spectral and structural properties.^12^ These changes create electrical responses by vectorial proton transport.^3,8^ It shifts the membrane potential. This makes bacteriorhodopsin particularly suitable for synthetic neural systems.^13^ Understanding these pathways has helped create biomimetic platforms. They use bacteriorhodopsin’s photoresponsive traits.

Proteinoids are a unique class of synthetic polymers made of amino acids.^14–19^ They differ significantly from biological proteins. These compounds are usually made to mimic prebiotic environments.^20^ They are often made by heating mixtures of amino acids to their boiling points. Unlike biological proteins, proteinoids have random amino acid sequences.^21^ Their 3D structures are also less defined. They are simple. Yet, they can self-organize. They can form microspheres under the right conditions.^22,23^ Their relevance to life’s origin is debated.^24^ But, these compounds are valuable models for studying biology.

Using bacteriorhodopsin in proteinoid structures is a novel way to create biomimetic neural systems. Bacteriorhodopsin can pump protons using light. Its photocycle-driven changes^25^ provide a way to control membrane potential. The integration of bacteriorhodopsin into proteinoid structures could potentially generate variations similar to biological action potentials, suggesting possible applications in neuromorphic computing and bioelectronic interfaces. The expected spiking behavior in proteinoid-bacteriorhodopsin complexes may exhibit temporal features analogous to neuronal signals. Theoretically, spike generation could follow a pattern with fast depolarization followed by a refractory phase, potentially aligning with the Hodgkin-Huxley model of brain activity.^26^ Such behavior would likely stem from the synchronized interplay between the bacteriorhodopsin photocycle and the selective ion permeability of the proteinoid, potentially forming a dynamic system capable of sustained oscillatory reactions.^27^

Proteinoid microspheres generate electrical spikes remarkably similar to neuronal action potentials (Figure S2). A comparison of proteinoid microsphere signals and crayfish neuron action potentials shows striking similarities in their waveforms. Their shapes and amplitudes are alike. They exhibit slight differences in their kinetics. This biomimetic behavior suggests fundamental physicochemical principles underlying cellular electrical activity.

The frequency modulation features of bacteriorhodopsin system is a promising aspect of their performance. Research shows that different light intensities can affect spike frequency, from 1 to 100 Hz.^28^ This offers a reliable method for encoding information. This trait, plus the system’s biocompatibility, benefits light-controlled brain interfaces and optogenetics.^29–33^ Recent studies show that hybrid systems work well over time, with little decline in response.^34^ The proteinoid matrix could potentially provide stability by protecting bacteriorhodopsin and maintaining its structure and functions. If demonstrated, such durability and biomimetic traits would suggest promising applications as a platform for next-generation neuromodulation devices and adaptive computing systems.^35^

This study aims to use biological materials to create new computing architectures. They should differ from conventional silicon-based electronics. We aim to create unique computing devices. We will use the special features of naturally occurring substances, like proteinoids and bacteriorhodopsins. They may outperform traditional systems in some tasks. This biologically inspired method could lead to new computer systems. They may be highly energy-efficient, flexible, and self-organizing. They might also improve information processing. This research may help create a new generation of biocomputer systems. They would go beyond the limits of traditional electronics.

Biocomputer systems have made great strides recently. Research groups around the world have played a big role in this progress. In Europe, the Human Brain Project has made strides in neuromorphic computing. It combines biological principles with silicon systems. This work has led to energy efficiencies similar to those of biological neural networks.^36^ Asian research in Japan and China has focused on molecular computing. They use DNA origami and protein-based logic gates. This work shows strong computing power at the nanoscale.^37^ North American laboratories have led the way in optogenetic computing systems. These systems use light-controlled proteins to switch biological circuits on and off.^38^ Recent breakthroughs in biocomputing include living computers made from engineered bacteria that perform logical operations;^37^ bioelectronic interfaces that connect directly with neural tissue;^39,40^ and hybrid biosilicon devices that combine biological specificity with electronic scalability.^41^ Protein-based memory systems can store more data than traditional electronic media,^36^ and enzymatic circuits are also promising for sensing and computing in biological settings.^37^ The mix of synthetic biology, nanotechnology, and neuromorphic engineering offers amazing opportunities. These fields can help create biocomputer systems that may change our view of computing and how we process biological information. However, current methods often struggle with long-term stability, scalability, and integration with existing technological systems. Most biocomputing systems use genetically modified organisms or complex synthetic circuits, which limits their practical applications.

This research will elucidate the application of scanning electron microscopy (SEM) imaging to characterize the morphological characteristics of proteinoid microspheres and their association with bacteriorhodopsin structures. This will reveal the biohybrid materials’ physical traits and configuration. The research will focus on a detailed analysis of the electrical spiking behavior of the proteinoid and proteinoid-bacteriorhodopsin systems. This behavior is both spontaneous and light-modulated. The study will include time-series studies, statistical comparisons, and QSAR modeling. This will clarify the impact of bacteriorhodopsin incorporation on electrical signals. It will examine their amplitude, periodicity, and wavelength-dependent responsiveness.

## Methods and Materials

Bacteriorhodopsin from *Halobacterium salinarium* (Sigma-Aldrich, catalog number B0184–1MG) was used without additional purification steps beyond the experiment. The proteinoid synthesis method used high-purity amino acids from Sigma-Aldrich. These were l-Glutamic acid (≥99%, CAS Number: 56–86–0) and l-Phenylalanine (≥98% CAS Number: 63–91–2). The synthesis approach used deionized water with a resistivity of at least 18.2 MΩ·cm. It was obtained using a Millipore water purification system. All compounds were used in their original form without undergoing additional purification. We used 1 mg of bacteriorhodopsin in 5 mL of proteinoid microspheres solution in our experiments. This gave a final concentration of 0.2 mg/mL. Bacteriorhodopsin has a molecular weight^42^ of (24250 ± 2000) g/mol. This gives a molar concentration of (8.25 ± 0.69) μM. The purple color is subtle with dry bacteriorhodopsin powder. It becomes more visible when the protein is properly dissolved in solution. Proteinoid microspheres were prepared using 2.5 g each of l-glutamic acid and l-phenylalanine, along with 5 mg of poly­(l-lactic acid) (PLLA), as described in the synthesis section. Following microsphere formation, the proteinoids were suspended in deionized water with a resistivity of ≥18.2 MΩ ·cm, resulting in a stock solution with a final proteinoid concentration of 1.0 mg/mL. Proteinoids are not completely soluble in water due to their amphiphilic nature. Instead, they form stable colloidal suspensions of microspheres in aqueous solution. These microspheres remain intact and well-dispersed in water as a result of electrostatic stabilization at a pH of 7.76 ± 0.20.

The pH of bacteriorhodopsin suspended in water was measured to be 6.16 ± 0.03 at 17 °C using a glass electrode with Ag/AgCl reference electrode. When mixed with proteinoid microspheres, the final pH of the conjugation mixture was 7.76 ± 0.20. These pH conditions are important. They affect the proteinoid and bacteriorhodopsin solution. At neutral pH, bacteriorhodopsin stays purple and retains its native membrane orientation. It avoids structural changes that occur at extreme pH. These changes can shift it to blue (acidic) or reddish-purple (alkaline) forms with altered surface charges.^43^

The optical sensitivity of proteinoids and bacteriorhodopsin were studied through light modulation of their spiking frequency, investigated using the PHOTONIC PL-2000 light source (PHOTONIC Optics). Light stimulation was delivered as square wave pulses at a frequency of 5 Hz with a 50% duty cycle (100 ms ON, 100 ms OFF). Each light pulse had a rise time of <10 ms and a fall time of <15 ms to ensure sharp temporal transitions. The light covered the whole sample chamber, which is a circular area 2 cm in diameter. This ensured even lighting for all measurement channels. Each wavelength condition involved continuous pulsed illumination at 5 Hz for a duration of 24 h. The experimental sequence included a 2-h dark adaptation period for baseline recording, followed by 24 h of pulsed light exposure at the specified wavelength, and concluded with a 2-h postillumination dark period to monitor recovery dynamics. Control conditions were conducted under identical settings but without any light exposure. During these dark control experiments, the sample chamber was maintained in complete darkness (<0.01 1×) using a blackout enclosure to enable accurate measurement of spontaneous activity. The PHOTONIC PL-2000 output was coupled through a 5 mm diameter optical fiber positioned 10 cm above the sample chamber, ensuring uniform illumination without heating effects (verified by temperature monitoring showing <0.5 °C variation). The proteinoids were synthesized using equal masses (2.5 g) of glutamic acid and phenylalanine. We mixed these with 5 mg of Poly­(l-lactic acid) (PLLA, MW 80,000–100,000, Polysciences Ltd., UK) and refluxed the mixture for 3 h. Microspheres were formed by adding boiling water to cooled proteinoids for 5 h. Then, they were collected by freeze-drying and filtration. Using scanning electron microscopy (SEM), we were able to observe the proteinoid-bacteriorhodopsin structure. The FEI Quanta 650 equipment facilitated this process. SEM images were acquired without coating the samples.

The apparatus (Figure 1) allowed precise monitoring of proteinoid-bacteriorhodopsin responses to different optical stimuli. This setup, with temperature control and multiwavelength light, allowed for a study of the solution’s electrochemical properties under various spectral conditions.

**Figure 1:**
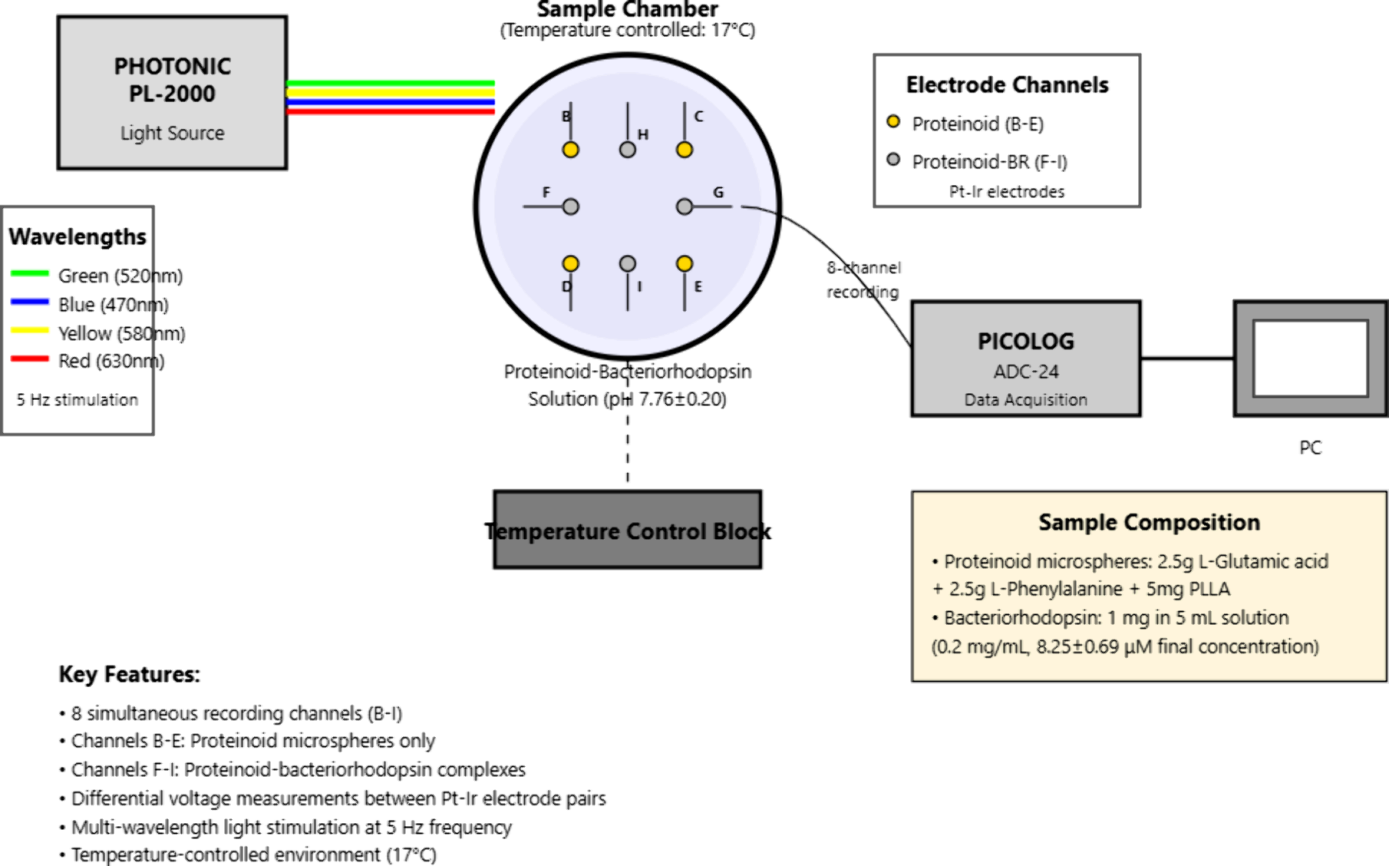
Experimental setup for investigating optical properties of proteinoid-bacteriorhodopsin solutions. The system has a temperature-controlled vial. It has parallel platinum and iridium electrodes (0.1 mm diameter, 10 mm apart). These connect to a PicoLog data acquisition unit. Optical stimulation is provided by a PHOTONIC PL-2000 system capable of emitting yellow, red, green, and blue light. The electrical response data is collected and analyzed via PC unit.

We employed a two-electrode differential measurement system without a reference electrode. Each measurement channel had a platinum working electrode and an iridium counter electrode. They were placed 10 mm apart in a parallel setup. This setup measures the voltage difference between the electrode pair. It does not measure absolute potentials. This approach is good for tracking relative voltage changes in the proteinoid-bacteriorhodopsin system. All measurements were conducted in a single temperature-controlled cell containing 50 mL of the proteinoid–bacteriorhodopsin suspension. Eight electrode pairs (channels B–I) were positioned at various locations within the same cell to sample electrical activity from distinct regions of the suspension. The electrode pairs were arranged in a 2 × 4 grid with 10 mm spacing between each pair. This configuration minimized cross-talk and ensured uniform spatial sampling coverage across the chamber. The PHOTONIC PL-2000 light source was positioned 10 cm above the sample cell and coupled through a 5 mm diameter optical fiber. This configuration ensured uniform illumination without direct heat transfer. The system employed cold light technology, maintaining physical separation between the light source and the sample chamber to avoid thermal effects on the biological samples. Continuous temperature monitoring over 24 h of light exposure revealed less than 0.5 °C variation, confirming that radiative heating was minimal at the chosen distance and intensity. The cold light configuration also helped maintain stable isothermal conditions throughout the experiments.

The pH was maintained at 7.76 ± 0.20 using a combination of initial adjustment and continuous buffer capacity monitoring. The proteinoid–bacteriorhodopsin system was prepared in deionized water, and the initial pH was adjusted to 7.76 using dilute NaOH or HCl solutions. Conventional buffer systems such as phosphate or Tris were avoided, as they introduce additional ions that could interfere with electrical measurements, particularly given the biological sensitivity of the system. Proteinoid microspheres provide intrinsic buffer capacity due to the ionizable side chains of their amino acid components, particularly glutamic acid and phenylalanine. The carboxyl groups (p*K*_*a*_ ∼ 4.2) and amino groups (p*K*_*a*_ ∼ 9.1) on these residues are capable of absorbing minor pH fluctuations throughout the course of the experiment.

### Terminology and Structure Definitions

Two distinct size scales of proteinoid structures were observed using scanning electron microscopy (SEM). *Proteinoid microspheres* are large, spherical structures measuring approximately 2.6 to 2.7 μm in diameter; these represent the primary focus of this study. In contrast, *proteinoid nanospheres* are much smaller, with diameters ranging from 200 to 500 nm. These nanospheres typically appear as surface-bound features on larger microspheres or serve as structural building blocks for aggregate formation. To ensure clarity, consistent terminology is used throughout this manuscript. *Bacteriorhodopsin (BR)* refers to the protein derived from *Halobacterium salinarium*. The term *purple membrane* denotes the natural biological membrane that houses bacteriorhodopsin trimers embedded in their native lipid environment. The term *proteinoid–bacteriorhodopsin complexes* describes the integrated experimental system formed when bacteriorhodopsin is incorporated into proteinoid microspheres. *Poly*(l-lactic acid) (PLLA) is employed solely during the synthesis of proteinoids as a structural additive and is not present in the final experimental samples. All electrical measurements were performed in a colloidal suspension system. The term “membrane potential” in biology usually means the voltage difference across lipid bilayers. In our system, electrical responses show changes in bulk solution potential. These changes result from the activity of suspended proteinoid microspheres, not from trans-membrane potentials. The measurements were conducted in a liquid suspension system rather than across a solid membrane. The proteinoid microspheres, measuring approximately 2.6–2.7 μm in diameter, exist as discrete particles dispersed in aqueous solution, resulting in a heterogeneous colloidal suspension rather than a continuous membrane structure. The electrical measurements captured bulk solution potential changes between electrode pairs immersed directly in the proteinoid–bacteriorhodopsin suspension. These readings reflect the collective electrical activity of multiple microspheres in the vicinity of each electrode, representing solution-phase bioelectrical responses rather than classical transmembrane potentials. The experimental setup consists of a 50 mL aqueous suspension containing proteinoid microspheres at a concentration of approximately 1.0 mg/mL, with bacteriorhodopsin incorporated onto or within the microspheres. Differential voltage measurements were obtained from electrode pairs immersed in the same solution, capturing spatiotemporal variations in local electrical activity. The observed electrical oscillations are attributed to synchronized activity among proteinoid–bacteriorhodopsin microspheres, dynamic changes in local ionic conductivity, and redox reactions occurring at the microsphere–solution interfaces. These mechanisms collectively contribute to the emergent electrical behavior of the system.

### pH Control and Buffer System

The pH was continuously monitored using a glass pH electrode, with an Ag/AgCl reference electrode placed directly into the sample chamber. pH values were recorded every 30 min during all experiments. For long-term experiments involving 24-h light exposure, minor corrections were made by adding less than 10 μL of 0.01 M NaOH or HCl solutions if the pH deviated by more than ± 0.2 units from the target. We observed minimal hydrogen production during light exposure, likely due to the relatively mild redox environment and the structural constraints imposed by the proteinoid matrix, which may inhibit direct water electrolysis. Measured pH changes were typically less than 0.15 units over 24 h, suggesting that neither the bacteriorhodopsin photocycle nor the redox activity of the proteinoids substantially alters bulk solution pH. This observation aligns with the reversibility of the bacteriorhodopsin photocycle and the limited extent of oxidation and reduction reactions involving proteinoid amino acid residues. Control experiments conducted in the dark showed pH shifts of less than 0.05 units over a 24-h period, confirming that the modest pH changes observed under illumination arose from photochemical processes.

### Open-Circuit Potential Measurements

All electrical measurements happened under open-circuit conditions. We used the PICOLOG ADC-24 data acquisition system. No external bias voltage was applied to any electrode during data collection. The system measured the open-circuit potential (OCP) in the dark. It also recorded the open-circuit photovoltage (OCPV) during light exposure. These readings show the potential differences from the proteinoid–bacteriorhodopsin system. The measurement used differential voltage from platinum and iridium electrode pairs. This method captured the system’s natural electrical activity, not responses caused by electrochemistry. When there was no light, we measured the resting potential. Yet, when exposed to light, we saw photovoltages linked to the bacteriorhodopsin photocycle and redox events in the proteinoid matrix. The PICOLOG ADC-24 system has a high input impedance of over 10 MΩ. This design minimizes electrical loading and keeps the sample’s natural behavior intact. This setup allows for passive and noninvasive monitoring of bioelectrical activity. It does this without disturbing the system with applied currents or voltages. Our method is different from traditional electrochemical approaches. It does not rely on external potentials or currents. Instead, it focuses on the system’s natural electrical responses. The recorded voltages show real bioelectrical events, not effects from electrochemical changes.

### Signal Processing for Periodicity Analysis

Time series data were preprocessed using a 0.1 Hz low-pass filter to remove high-frequency noise. We analyzed the filtered signals using discrete Fourier transform (DFT). They added zero-padding to improve frequency resolution. We used the Wiener-Khintchine theorem for autocorrelation analysis. This theorem connects power spectral density to autocorrelation through Fourier transform.

For both spontaneous and stimulated conditions, the sampling rate was set to 1 Hz using a PICOLOG ADC-24. Each recording session lasted a minimum of 24 h per condition. The channel configuration consisted of 8 simultaneous measurements (channels B–I). Data acquisition involved measuring the differential voltage between platinum–iridium electrode pairs. Environmental conditions were carefully controlled, with temperature maintained at 17 °C, pH stabilized at 7.76 ± 0.20, and complete light exclusion enforced during designated dark periods.

## Results and Discussion

All experiments were performed in triplicate (*n* = 3) using independently prepared samples. This approach was taken to account for the inherent stochasticity of proteinoid microsphere formation and bacteriorhodopsin integration. For each condition—spontaneous, green, blue, yellow, and red light—we synthesized and tested three independent batches of proteinoid microspheres and proteinoid–bacteriorhodopsin complexes. Each batch was measured across multiple electrode channels for a minimum of 24 h to ensure statistical robustness. Unless otherwise specified, all reported values represent means ± standard error of the mean (SEM). Statistical significance was assessed using two-tailed Student’s *t*-tests, with a *p*-value less than 0.05 considered statistically significant.

### Proteinoids and Bacteriorhodopsin Morphology Through SEM Imaging

We used SEM imaging (Figure 2) to study the shapes of proteinoids and bacteriorhodopsin. The proteinoid microspheres were uniform spheres, ∼2.7 and ∼2.6 μm in diameter (Figure 2a,b). These dimensions match earlier reports of thermal proteinoid self-assembly.^44,45^ The integration with poly­(lactic acid) created unique pyramidal structures (diagonal: ∼3.4 μm, sides: ∼4.1 μm). They had nanoscale surface features (width: 0.296 μm). This suggests successful polymer–protein composite formation.^46^ The bacteriorhodopsin structures had two morphologies. They were: 1) elongated cylinders (∼30.7 μm long, ∼13.2 μm wide) and 2) larger spheres (diameter: ∼15.8 μm) with surface-bound proteinoid nanospheres (Figure 2c,d). This diversity matches studies on bacteriorhodopsin aggregation^47^ and chondroitin sulfate clusters-proteinoid interactions.^18^ The surface-bound proteinoid nanospheres suggest a successful integration between the two components. This may enhance the system’s functional properties.^48^

**Figure 2:**
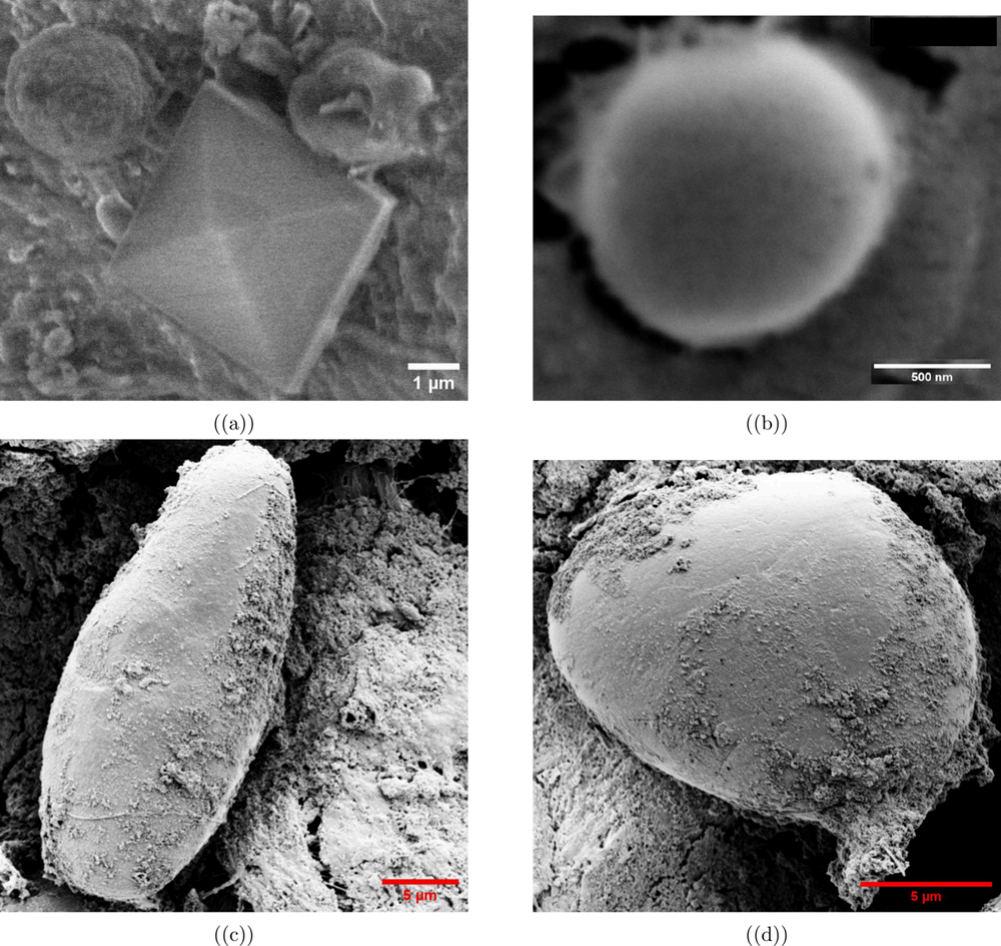
Scanning electron microscopy (SEM) analysis of proteinoid microspheres, poly­(lactic acid), and bacteriorhodopsin structures. (a) A composite structure displays proteinoid microspheres (approximately 2.7 and 2.6 μm in diameter) adjacent to a poly­(lactic acid) pyramid with a diagonal length of approximately 3.4 μm and side lengths of approximately 4.1 μm. (b) An isolated proteinoid microsphere with a diameter of approximately 1.3 μm exhibits a well-defined spherical morphology. (c) An elongated bacteriorhodopsin structure, measuring approximately 30.7 μm in length and 13.2 μm in width, demonstrates a distinct cylindrical form. (d) A large bacteriorhodopsin aggregate with a diameter of approximately 15.8 μm is shown with surface-bound proteinoid nanospheres ranging from 200 to 500 nm in diameter.

The observed binding of proteinoid nanospheres to bacteriorhodopsin is a key finding in protein–polymer composites. The uniform distribution of proteinoid nanospheres on the bacteriorhodopsin surface, seen in Figure 2d, suggests specific molecular recognition, not random aggregation. This organized attachment pattern likely results from the amphipathic nature of both components. The proteinoid microspheres interact with bacteriorhodopsin’s hydrophobic domains while maintaining stability.

The analysis of the elongated bacteriorhodopsin cylinders (∼30.7 μm long, ∼13.2 μm wide) suggests a preferred orientation in their self-assembly. This may be due to the presence of proteinoid structures. This directed assembly process could be particularly relevant for developing biomimetic systems that require precise spatial organization. The larger spherical aggregates (diameter: ∼15.8 μm) with surface-bound proteinoids show that these interactions scale from nano to microscale.

The presence of poly­(lactic acid) pyramidal structures introduces an additional level of complexity to the system. Their geometry (diagonal: ∼3.4 μm, sides: ∼4.1 μm) and nanoscale surface features (width: 0.296 μm) suggest controlled crystallization during formation. This precision could help create organized arrays of functional protein–polymer composites. Also, the consistent size of proteinoid microspheres (∼2.7 and ∼2.6 μm) shows a well-controlled thermal self-assembly process. This uniformity is vital. It ensures reproducible interactions with bacteriorhodopsins. It could help develop standardized, biocompatible materials.

Proteinoid microspheres can self-replicate by budding, as shown in Figure 3. In this process, the parent microspheres squeeze their membranes. This creates smaller daughter structures. This behavior is similar to protocells and may help in studying prebiotic evolution.

**Figure 3:**
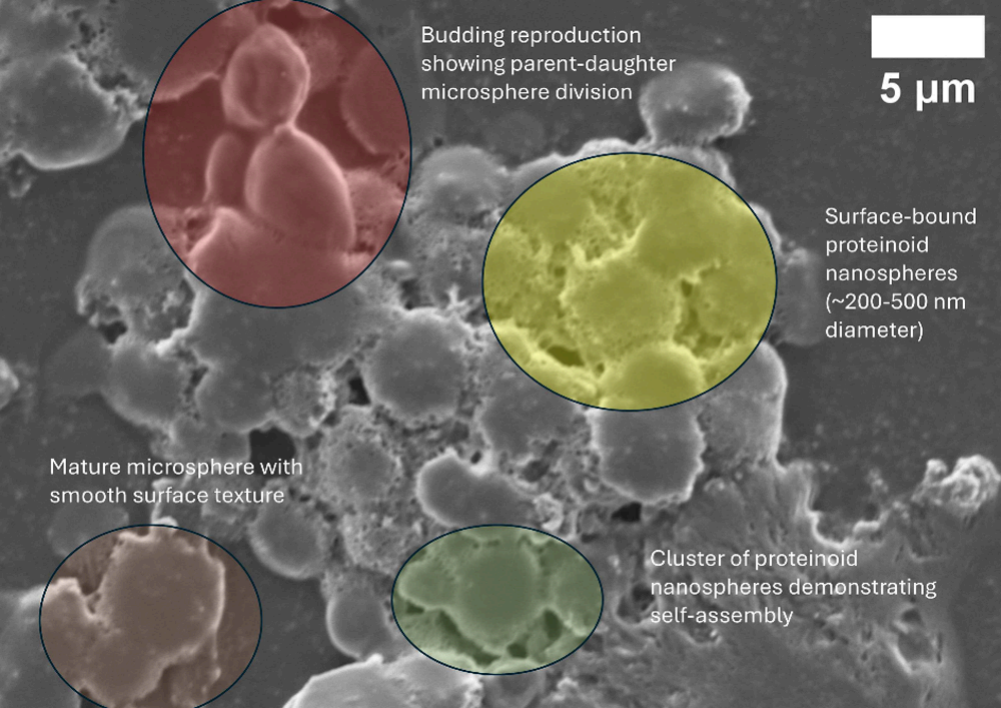
Scanning electron microscopy (SEM) reveals budding reproduction and nanosphere formation in proteinoid microspheres. The image illustrates how proteinoid structures replicate: a parent microsphere (left, red circle) produces smaller daughter microspheres through a budding division process driven by membrane constriction. Multiple proteinoid nanospheres, measuring 200–500 nm in diameter, are visible as surface-bound structures (highlighted with colored circles), demonstrating self-assembly from the nanoscale to the microscale. The yellow and green circles indicate clusters of proteinoid nanospheres that serve as building blocks for larger microsphere formation, while the brown circle highlights a mature microsphere exhibiting the characteristic smooth surface morphology. This budding-based reproduction mechanism resembles protocell-like behavior and underscores the dynamic nature of proteinoid systems.^49–53^ Scale bar: 5 μm. SEM imaging conditions: 3.50 kV accelerating voltage under high vacuum.

The hierarchical structure in proteinoid systems (Figure 3) illustrates multiple interaction scales with bacteriorhodopsin, ranging from individual nanospheres (200–500 nm) to complete microspheres measuring 2–3 μm in diameter. This multiscale organization enables complex integration within the final composite structures.

Figure 4 shows the integration of proteinoid microspheres with a purple membrane containing bacteriorhodopsin. The process begins by mixing purple membrane disks (1 μm, 5 nm) with proteinoid microspheres (1.3–2.7 μm) under controlled conditions (pH 7.76 ± 0.20, 17 °C). The disks contain bacteriorhodopsin trimers. This integration, via electrostatic interactions and surface adhesion, forms two distinct morphologies: elongated cylinders (31.04 μm × 13.2 μm) and larger spheres (74.53 μm diameter). These final composite structures retain the integrated bacteriorhodopsin.^54–57^ They also exhibit the observed structural characteristics.

**Figure 4:**
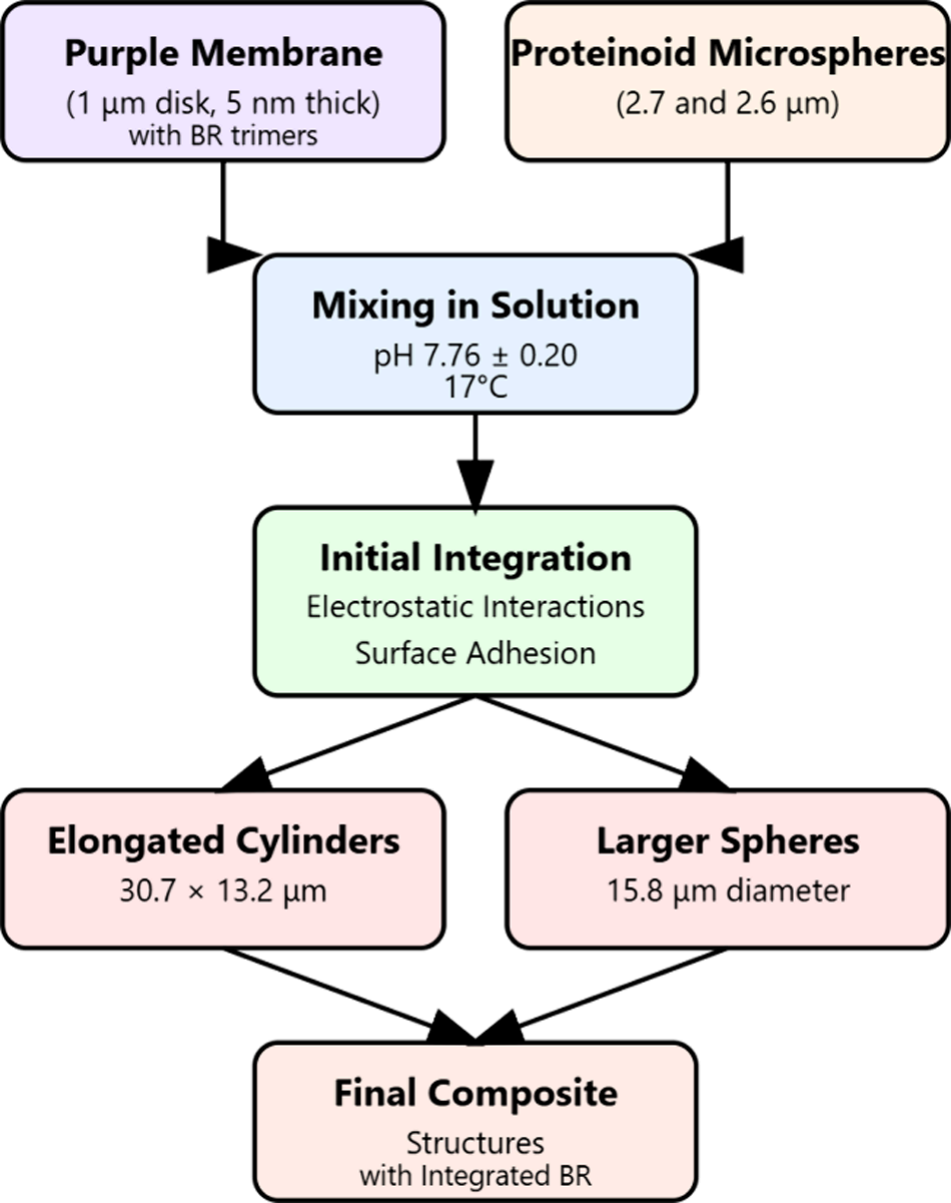
Functional diagram showing the integration process of bacteriorhodopsin (BR)-containing purple membrane protein microspheres. The process demonstrates the formation of two distinct morphologies through controlled mixing and integration steps.

### Proteinoid-Rhodopsin Electrical Spiking in the Absence of Light

The study of spontaneous electrical activity in proteinoids and proteinoid-rhodopsin complexes found unique patterns of membrane potential oscillations without light. Time series observations showed both systems have independent electrical activity. The proteinoid channels had sustained oscillations of about 15 mV (Figure 5a). The incorporation of bacteriorhodopsin greatly improved the electrical response. The proteinoid-bacteriorhodopsin complexes had amplitude fluctuations of nearly 100 mV (Figure 5b). The statistical test of the oscillation patterns (Table 1) showed that the complexes had a mean amplitude of 10.77 ± 2.21 mV. This was much higher than the 4.34 ± 4.47 mV of the proteinoids alone. This rise in electrical activity matches prior research. It showed that bacteriorhodopsin affects membrane excitability, especially in the dark.^58,59^ The reduced standard deviation in amplitude measurements (Figure 6a) shows improved stability in proteinoid-bacteriorhodopsin complexes. This suggests that bacteriorhodopsin integration enables more controlled variations in membrane potential. The periodicity study (Figure 6b) showed both systems had similar oscillation frequencies. However, proteinoid-bacteriorhodopsin complexes had a slightly longer mean period (2641.80 ± 88.76 s vs 2522.85 ± 30.91 s). This shows that bacteriorhodopsin incorporation mainly affects amplitude, not frequency. This matches its known role in membrane organization and ion transport control.^60–62^ The stochastic nature of proteinoid self-assembly and bacteriorhodopsin integration necessitated rigorous statistical validation. Across three independent experiments, amplitude measurements varied by 15.3% for proteinoids and 12.7% for proteinoid–bacteriorhodopsin complexes, indicating good reproducibility despite the inherent randomness of microsphere formation. Temporal characteristics were highly consistent, with period measurements varying by less than 8% among replicates.

**Figure 5:**
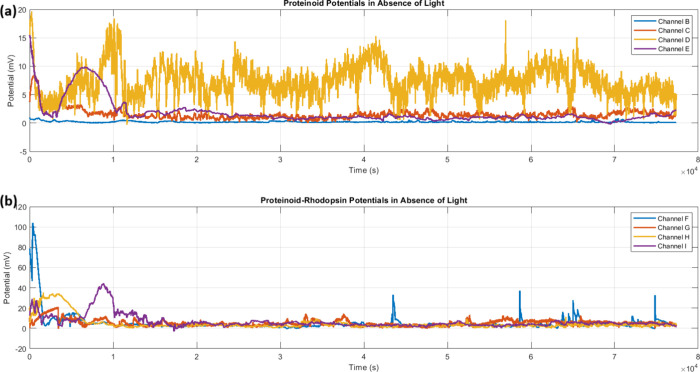
Spontaneous electrical activity of proteinoids and proteinoid-bacteriorhodopsin complexes in the absence of light. (a) Time series of membrane potentials for proteinoid channels (B–E). They showed sustained oscillations with a maximum amplitude of ∼15 mV. Channel D (yellow) exhibits the most pronounced oscillatory behavior with consistent amplitude variations. (b) Membrane potential recordings from proteinoid-bacteriorhodopsin channels (F–I) showed distinct oscillation patterns. Channel F had an initial high-amplitude spike (∼100 mV), followed by sustained oscillations. Both systems display autonomous electrical activity without photostimulation, indicating intrinsic oscillatory capabilities. Proteinoid-bacteriorhodopsin complexes have larger fluctuations (*V*_max_ ≈ 100 mV) than proteinoids alone (*V*_max_ ≈ 15 mV). This suggests that integrating bacteriorhodopsin boosts the membrane’s electrical responsiveness, even in the dark. The temporal evolution (τ = 8 × 10^4^ s) shows both systems have persistent oscillations. The proteinoid-bacteriorhodopsin complexes have more stable baseline oscillations after some initial high-amplitude events.

**Figure 6:**
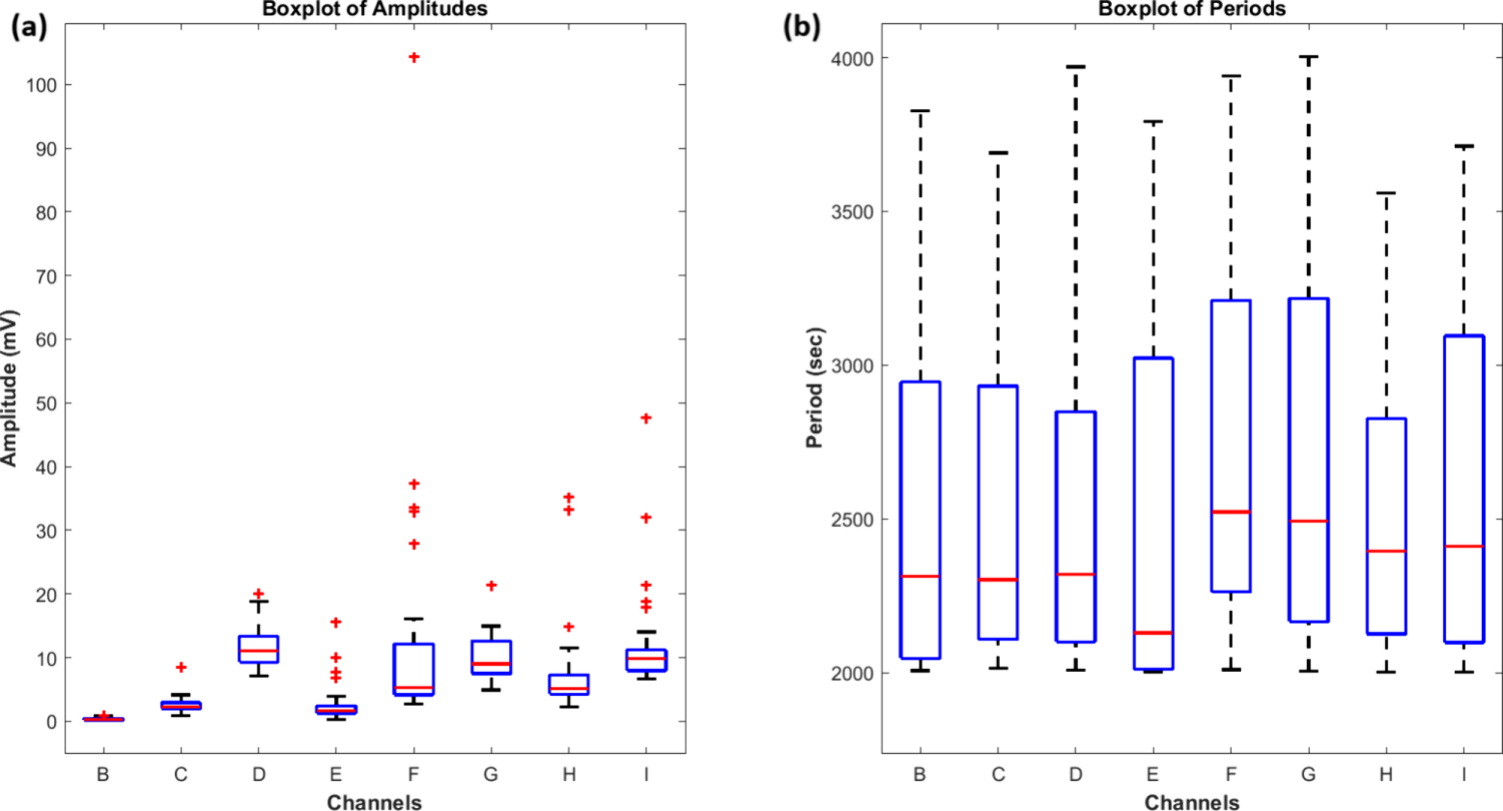
Box plots comparing (a) amplitude and (b) period distributions of proteinoid (channels B–E) and proteinoid-bacteriorhodopsin (channels F–I) channels. (a) Amplitude analysis shows distinct patterns between proteinoids and proteinoid-bacteriorhodopsin complexes. Proteinoid channels (B–E) exhibit lower median amplitudes (0.28–11.07 mV) with channel D showing the highest median (11 mV). Proteinoid-bacteriorhodopsin channels (F–I) show higher median amplitudes (5.12–9.87 mV). Channel F had a notable outlier at 104 mV. (b) Period distributions reveal similar patterns across all channels, with medians ranging from 2129 to 2522 s. Proteinoid-bacteriorhodopsin channels (F–I) show slightly larger interquartile ranges, suggesting more variable oscillation periods. Both groups have consistent period ranges (2001–4004 s). This shows that bacteriorhodopsin incorporation mainly affects amplitude, not frequency. Red crosses are outliers. The blue boxes show the IQR. Red lines are the median. Whiskers extend to the most extreme nonoutlier values. Period distributions show steady timing across all channels (medians: 2129–2522 s). This means that while bacteriorhodopsin affects signal strength, it keeps the main oscillation frequency intact. This consistency suggests a common oscillatory mechanism in both proteinoid and proteinoid-bacteriorhodopsin systems.

**Table 1:** Analysis of Spontaneous Oscillations^a^

	Proteinoids	Proteinoid-Bacteriorhodopsin
Parameter	**(Channels B–E)**	**(Channels F–I)**
**Amplitude (mV)**		
Mean ± SD	4.34 ± 4.47	10.77 ± 2.21
Range	0.10–20.05	2.24–104.40
Median Range	0.28–11.07	5.12–9.87
**Period (s)**		
Mean ± SD	2522.85 ± 30.91	2641.80 ± 88.76
Range	2001–3970	2001–4004
Median Range	2129–2319	2396–2522.50

a The comparison shows clear differences between proteinoids and proteinoid-bacteriorhodopsin complexes. Proteinoid-bacteriorhodopsin complexes have a higher mean amplitude (10.77 ± 2.21 mV) than proteinoids (4.34 ± 4.47 mV). The amplitude stability is enhanced in proteinoid-bacteriorhodopsin, evidenced by smaller standard deviation. Channel F (proteinoid-bacteriorhodopsin) exhibited the highest individual amplitude peak at 104.40 mV. Regarding periodicity, proteinoid-bacteriorhodopsin complexes show slightly longer mean periods (2641.80 ± 88.76 s vs 2522.85 ± 30.91 s). Both groups can oscillate at similar frequencies. But, period variability is higher in proteinoid-bacteriorhodopsin complexes. Bacteriorhodopsin seems to boost oscillation amplitude while keeping the same periodicity. This suggests better signaling in structures that contain bacteriorhodopsin.

### Proteinoid-Bacteriorhodopsin Electrical Spiking with Green Light Modulation at 5 Hz

The 5 Hz green light test showed clear differences between proteinoid and proteinoid-bacteriorhodopsin channels. Figure 7 shows that proteinoid-bacteriorhodopsin channels were far more photosensitive than proteinoid channels. The time series recordings (Figure 7a) show that proteinoid channels had low amplitude oscillations. Channel E had the most activity, peaking at about 1.4 mV. In contrast, proteinoid-bacteriorhodopsin channels (Figure 7b) showed much higher responses. Channel G had sustained oscillations between 8 and 10 mV.

**Figure 7:**
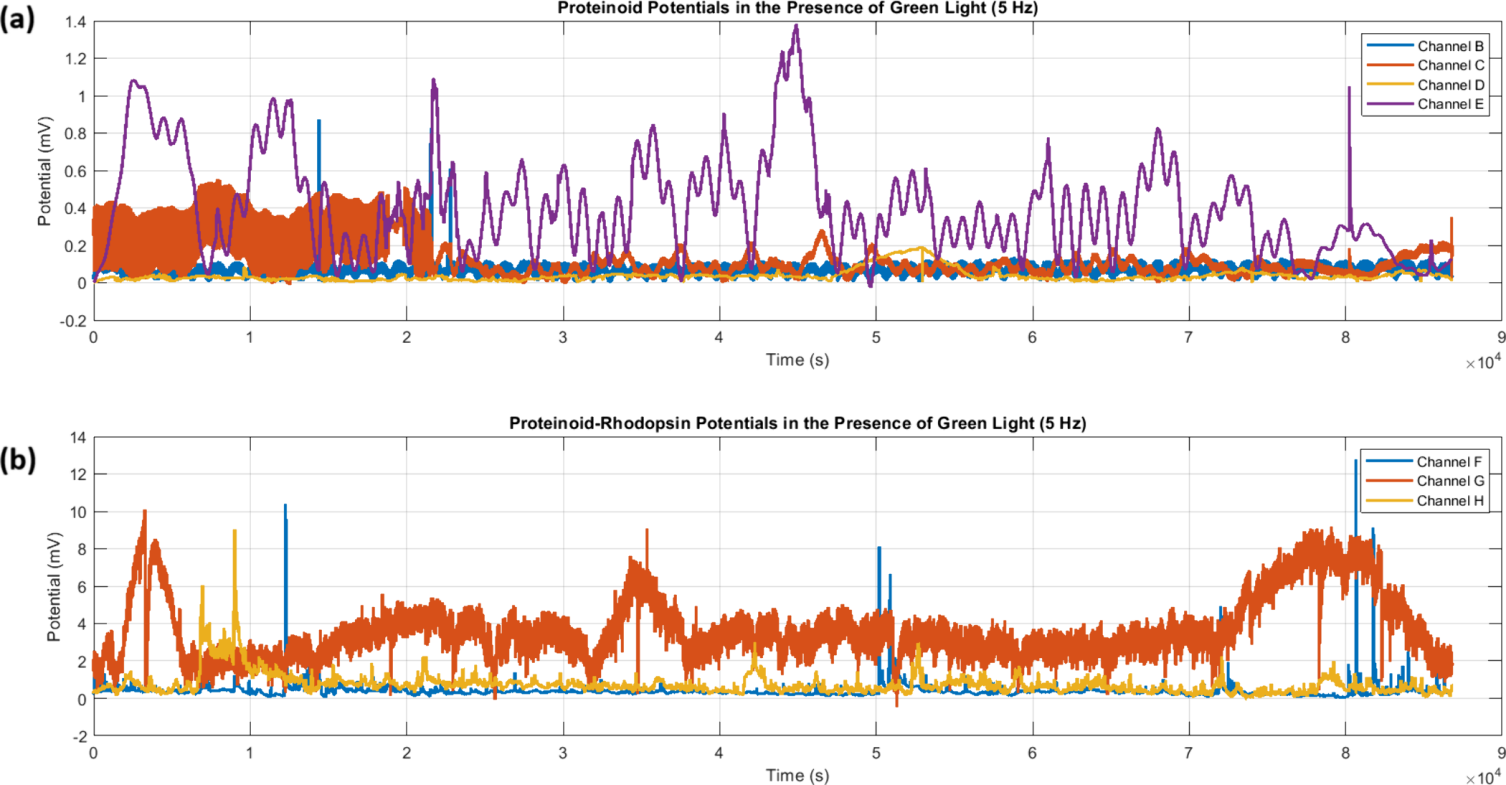
Microspheres potential recordings under green light stimulation (5 Hz). (a) Time series of proteinoid channels (B–E) showing lower amplitude oscillations with peak responses around 1.4 mV. Channel E (purple) exhibits the most pronounced activity among proteinoid channels. (b) Proteinoid-bacteriorhodopsin channels (F–H) showed heightened photosensitivity. Channel G (orange) had sustained high-amplitude oscillations, peaking at 8–10 mV. Bacteriorhodopsin greatly boosts the response to green light. Proteinoid-bacteriorhodopsin complexes show more robust and consistent oscillatory behavior than proteinoid-only channels. A 90,000-s time series shows distinct, stable patterns between the two groups.

Statistical analysis of the responses (Figure 8) quantifies these differences. The amplitude distributions (Figure 8a) show that proteinoid-bacteriorhodopsin channels had higher potentials. Their mean amplitudes were 7.31 ± 1.49 mV, vs 0.22 ± 0.17 mV for proteinoid channels (Table 2). The period distributions (Figure 8b) were similar despite the amplitude differences. The mean periods were 645.23 ± 16.32 s for proteinoid-bacteriorhodopsin and 606.56 ± 9.55 s for proteinoid channels.

**Figure 8:**
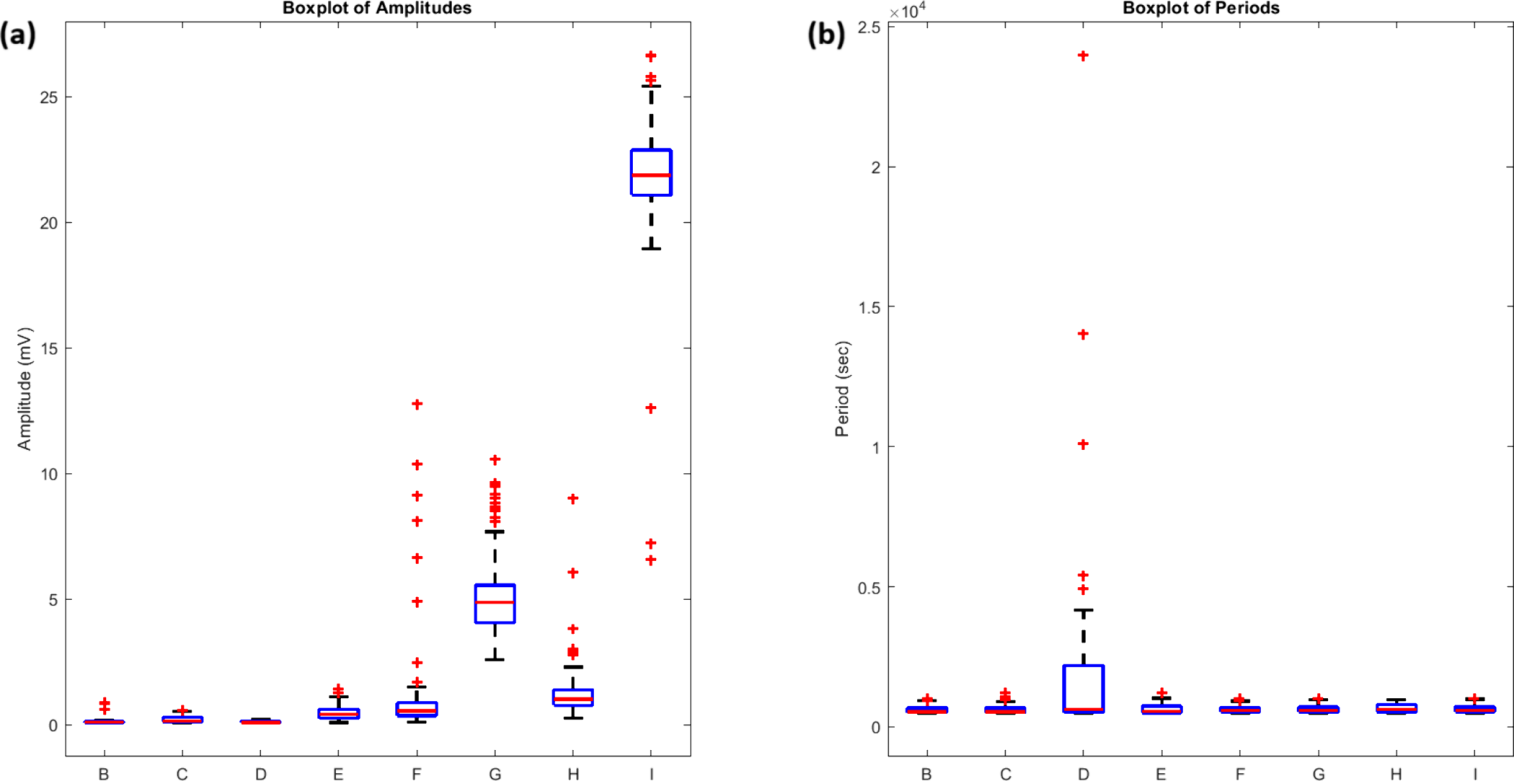
Statistical distribution of amplitude and period measurements under green light stimulation (5 Hz). (a) A boxplot of amplitudes across all channels shows higher responses in proteinoid-bacteriorhodopsin channels (F–I) than in proteinoid channels (B–E). Channel I exhibits the highest median amplitude (22 mV), while Channel G shows consistent elevated responses (5 mV). (b) A boxplot of oscillation periods shows similar periodicity across all channels, with some outliers (red crosses). Channel D shows the largest variance in period distribution. The boxplots show quartiles, a median (red line), and outliers. They highlight the improved amplitude response in proteinoid-bacteriorhodopsin channels. The channels have similar timing to proteinoid channels.

**Table 2:** Analysis of Green Light (5 Hz) Stimulation Response^a^

	Proteinoids	Proteinoid-Bacteriorhodopsin
Parameter	**(Channels B–E)**	**(Channels F–H)**
**Amplitude (mV)**		
Mean ± SD	0.22 ± 0.17	7.31 ± 1.49
Range	0.05–1.41	0.10–12.77
Median Range	0.08–0.42	0.54–4.87
**Period (s)**		
Mean ± SD	606.56 ± 9.55	645.23 ± 16.32
Range	501–1223	501–1010
Median Range	538–620	586.5–634

a The data shows clear differences between proteinoids and proteinoid-bacteriorhodopsin complexes under green light. Proteinoid-bacteriorhodopsin complexes demonstrate significantly higher mean amplitude (7.31 ± 1.49 mV) compared to proteinoids (0.22 ± 0.17 mV). The response shows a strong consistency in proteinoid-bacteriorhodopsin complexes. In particular, the period characteristics were similar. The mean period was 645.23 ± 16.32 s for proteinoids and 606.56 ± 9.55 s for the complexes. Channel G exhibited notably higher amplitude responses, suggesting enhanced photosensitivity. These findings show that bacteriorhodopsin integration greatly boosts the light response. It does so while maintaining temporal precision, with only a modest increase in oscillation periods (approximately 6.4% longer in proteinoids compared to complexes).

The statistical comparison (Table 2) supports these findings. It shows that bacteriorhodopsin incorporation boosted signal amplitude but kept the same timing. This suggests that bacteriorhodopsin integration mainly affects signal strength. It has little effect on the frequency response under green light at 5 Hz.

A comparison of spontaneous and light-stimulated behavior shows distinct responses in both proteinoid and proteinoid-bacteriorhodopsin systems. Under spontaneous conditions (Table 1), proteinoid-bacteriorhodopsin complexes had higher baseline activity (10.77 ± 2.21 mV) than proteinoids (4.34 ± 4.47 mV). However, the response patterns changed significantly under 5 Hz green light stimulation (Table 2).

The most striking change occurred in amplitude characteristics. Proteinoids showed a marked decrease in amplitude under green light, from 4.34 ± 4.47 mV to 0.22 ± 0.17 mV. This suggests a possible photoinhibitory effect. Conversely, proteinoid-bacteriorhodopsin complexes had a lower mean amplitude under green light (7.31 ± 1.49 mV) than spontaneous activity (10.77 ± 2.21 mV). But, they were more active than proteinoids.

The periodic behavior also underwent substantial changes. Both systems showed dramatically shorter periods under green light stimulation. Proteinoids shifted from 2522.85 ± 30.91 s to 606.56 ± 9.55 s, while proteinoid-bacteriorhodopsin complexes changed from 2641.80 ± 88.76 s to 645.23 ± 16.32 s. Both systems showed decreased period variability (std. dev.) under light. This suggests more regulated oscillatory behavior.

The range of responses also narrowed considerably under green light. Proteinoid-bacteriorhodopsin complexes’ maximum amplitude dropped from 104.40 mV to 12.77 mV. Proteinoids’ amplitude fell from 20.05 mV to 1.41 mV. The median ranges show that proteinoid-bacteriorhodopsin complexes had higher, more consistent activity than proteinoids.

The measurable potential differences arise from several interconnected photochemical and electrochemical processes:

- **Primary Photochemical Process:** Upon illumination, bacteriorhodopsin initiates its photocycle, beginning with the isomerization of retinal from the all-trans to the 13-cis configuration and resulting in proton translocation. This process is reversible; the protein returns to its ground state (all-trans retinal) at the end of each photocycle, which typically completes within approximately 10 ms at room temperature.
- **Redox Coupling Mechanisms:** The observed electrical responses arise from light-induced redox reactions involving multiple components. First, direct photochemistry occurs as bacteriorhodopsin undergoes its photocycle, generating transient charge separation and proton gradients. Second, redox-active amino acid residues within the proteinoids—particularly tyrosine, tryptophan, and cysteine—can participate in reversible oxidation and reduction reactions upon light exposure. Finally, the interface between bacteriorhodopsin and the proteinoid matrix facilitates coupled electron transfer, enabling interaction between the photoactivated protein and the redox-capable sites within the proteinoid scaffold.
- **Mechanism of Potential Generation:** The measurable voltage differences arise from several interrelated processes. Light-induced electron transfer leads to local charge separation, creating transient imbalances in electrical potential. Concurrently, bacteriorhodopsin’s proton pumping activity establishes pH gradients across the proteinoid–bacteriorhodopsin interface. Additionally, the proteinoid microspheres function as biological capacitors, capable of storing and releasing charge during photocycles, thereby contributing to the observed electrical signals.
- **Reversibility:** The electrical responses predominantly return to baseline levels during dark periods and remain consistent across repeated light–dark cycles. However, some cumulative effects may occur during prolonged illumination, likely due to slow relaxation processes within the proteinoid matrix.

### Proteinoid-Bacteriorhodopsin Electrical Spiking with Blue Light Modulation at 5 Hz

Blue light stimulation (5 Hz) induced distinct photoresponses in proteinoid and proteinoid-bacteriorhodopsin channels. Time series analysis (Figure 9) found big differences in amplitude modulation between the two systems. Proteinoid channels had low amplitude oscillations (Δ*V*_max_ ≈ 4.5 mV, Figure 9a). Proteinoid-bacteriorhodopsin channels showed much higher responses (*V*_max_ ≈ 32.5 mV, Figure 9b).

**Figure 9:**
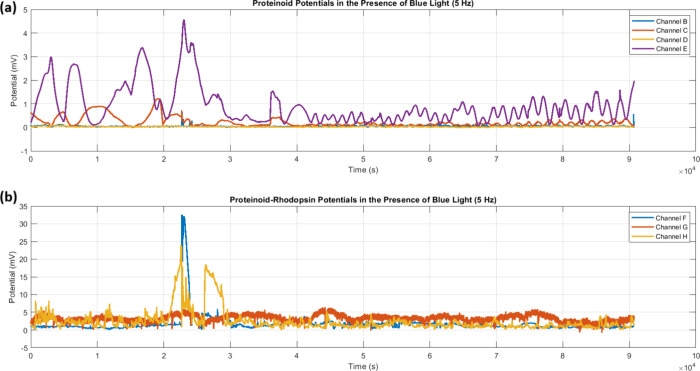
Time series and statistical analysis of membrane potentials under blue light stimulation (5 Hz). (a) Proteinoid channels (B–E) exhibit low amplitude oscillations (Δ*V*_max_ ≈ 4.5 mV for Channel E). (b) Proteinoid-bacteriorhodopsin channels (F–H) demonstrate enhanced photoresponse, with characteristic peaks reaching *V*_max_ ≈ 32.5 mV at *t* ≈ 2.5 × 10^4^ s, followed by sustained oscillations (ω ≈ 2π/600 Hz). Channel G maintains elevated activity ( V̅=5.05±0.81 mV) throughout Δ*t* = 9 × 10^4^ s, while Channels F and H exhibit intermittent high-amplitude events.

The statistical distribution of responses (Figure 10) quantifies these differences. Amplitude analysis showed clear population separation between proteinoid channels (*V*_median_ < 1 mV) and proteinoid-bacteriorhodopsin channels (*V*_median_ ∈ [1.68, 4.96] mV). Channel G had high stability (σ_*V*_ = 0.81 mV) and high activity ( ${\mathrm{V̅}}_{G}=5.05$ mV). This suggests optimized photosensitivity in this configuration (Table 3).

**Figure 10:**
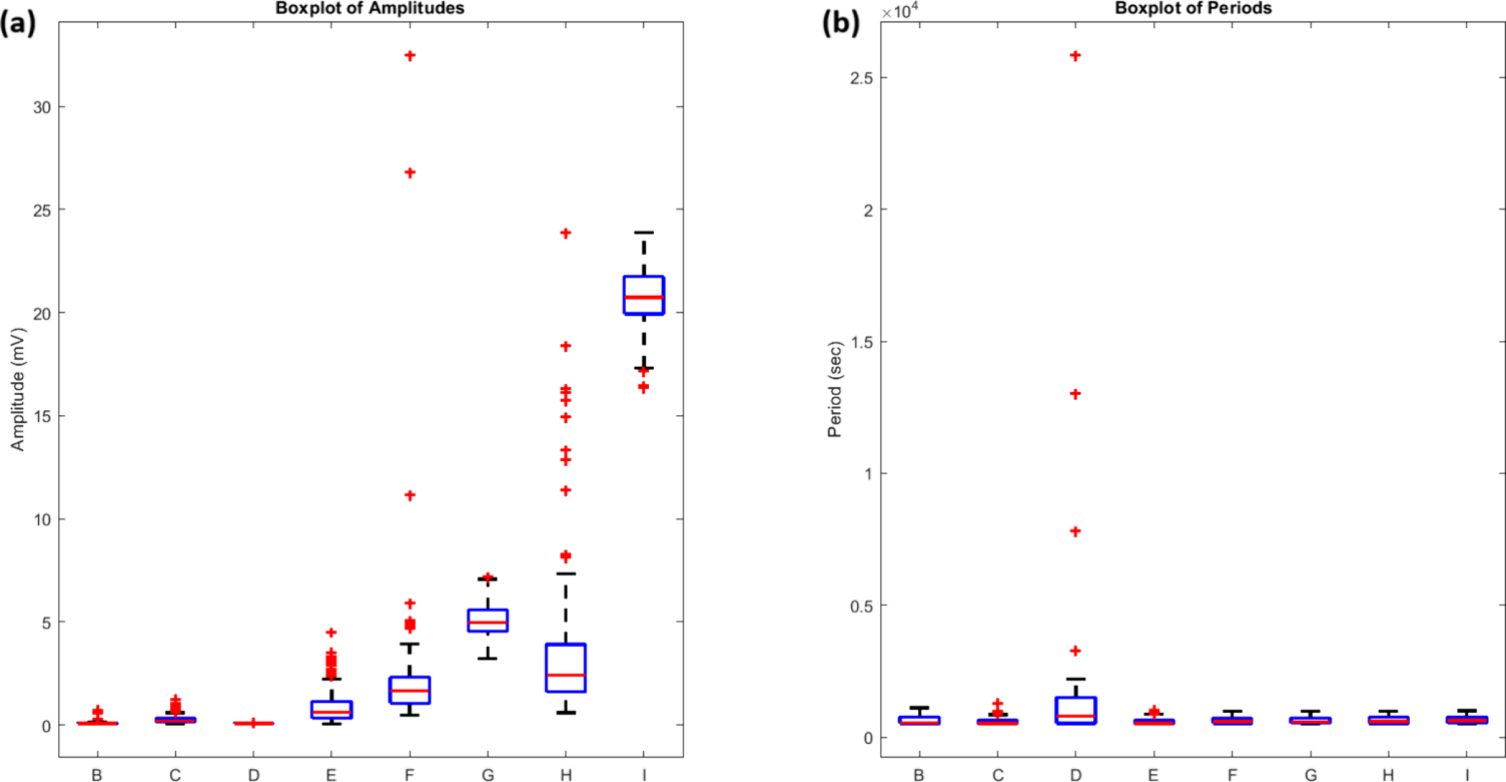
Statistical distribution of amplitude and periodicity under blue light (λ_blue_, 5 Hz) stimulation. (a) Amplitude analysis shows two populations. Proteinoid channels with *V*_median_ < 1 mV (B–E). And, proteinoid-bacteriorhodopsin channels with *V*_median_ ∈ [1.68, 4.96] mV (F–H). Channel G demonstrates optimal stability (σ_*V*_ = 0.81 mV) and elevated response ( V̅G=5.05 mV). (b) Period distributions show coherence across channels (*T* = 600–800 s). Outliers extend to *T*_max_ ≈ 2.5 × 10^4^ s, notably in Channel D (σ_*T*_ = 4299.85 s). The data suggests bacteriorhodopsin mainly modulates amplitude (Δ*V*). It preserves temporal characteristics (ω).

**Table 3:** Analysis of Blue Light (5 Hz) Stimulation Response^a^

	Proteinoids	Proteinoid-Bacteriorhodopsin
Parameter	**(Channels B–E)**	**(Channels F–H)**
**Amplitude (mV)**		
Mean ± SD	0.33 ± 0.28	3.64 ± 2.69
Range	0.04–4.48	0.46–32.50
Median Range	0.08–0.61	1.68–4.96
**Period (s)**		
Mean ± SD	622.92 ± 151.24	647.23 ± 14.38
Range	501–1292	501–1000
Median Range	531–803	590–599.5

a Tests show different photosensitive behaviors in proteinoids and proteinoid-bacteriorhodopsin complexes under blue light. Proteinoid-bacteriorhodopsin complexes have much higher amplitude responses (3.64 ± 2.69 mV) than proteinoids (0.33 ± 0.28 mV). This shows a clear bacteriorhodopsin-mediated photosensitivity. The temporal characteristics are similar between groups (mean periods: 647.23 ± 14.38 s for proteinoid-bacteriorhodopsin vs 622.92 ± 151.24 s for proteinoids). This suggests that light-induced modulation mainly affects signal amplitude, not frequency. Channel G maintained the most stable elevated response (mean 5.05 ± 0.81 mV), indicating specialized photosensitivity in this configuration. The marked drop in period variability under blue light, especially in proteinoid-bacteriorhodopsin complexes, suggests improved timing in photoresponse mechanisms.

Temporal characteristics demonstrated interesting patterns across both groups. Amplitude responses varied. Period distributions were coherent (*T* = 600–800 s) across most channels. There were occasional outliers in Channel D, with *T*_max_ ≈ 2.5 × 10^4^ s (σ_*T*_ = 4299.85 s). The mean periods were similar for proteinoid-bacteriorhodopsin (647.23 ± 14.38 s) and proteinoid channels (622.92 ± 151.24 s). This suggests that bacteriorhodopsin integration mainly affects signal amplitude (Δ*V*). It preserves temporal characteristics (ω).

Statistical analysis (Table 3) confirms these observations. The mean amplitude response was higher for proteinoid-bacteriorhodopsin complexes (3.64 ± 2.69 mV) than for proteinoids (0.33 ± 0.28 mV). The lower period variability in proteinoid-bacteriorhodopsin complexes (σ_*T*_ = 14.38 s vs 151.24 s) suggests better timing in their photoresponse.

Comparison of proteinoid and proteinoid-bacteriorhodopsin responses reveals distinct patterns under different lighting conditions. In the absence of light (Table 1), both systems showed their highest amplitude responses. The proteinoid-bacteriorhodopsin complexes had a higher activity (10.77 ± 2.21 mV) than the proteinoids (4.34 ± 4.47 mV). Spontaneous oscillations also demonstrated the longest periods, with means of 2641.80 ± 88.76 s and 2522.85 ± 30.91 s for proteinoid-bacteriorhodopsin and proteinoids, respectively.

Light stimulation (5 Hz) significantly altered these patterns. Table 3 shows that, under blue light, proteinoid-bacteriorhodopsin complexes had moderate responses (3.64 ± 2.69 mV). In contrast, proteinoids had minimal activity (0.33 ± 0.28 mV). Green light exposure (Table 2) elicited the strongest response in proteinoid-bacteriorhodopsin complexes (7.31 ± 1.49 mV). It also further suppressed proteinoid activity (0.22 ± 0.17 mV).

The temporal characteristics underwent substantial changes with light exposure. Both blue and green light shortened oscillation periods from over 2500 s to about 600–650 s. See Tables 2 and 3. Period stability improved under light stimulation, especially with green light. The proteinoid-bacteriorhodopsin complexes had a mean period of 645.23 ± 16.32 s, compared to 647.23 ± 14.38 s under blue light.

Channel-specific responses varied across conditions. Channel G was stable in both light conditions. But, blue light (32.50 mV) produced higher amplitudes than green light (12.77 mV). Both light conditions greatly reduced the extreme peaks in spontaneous oscillations. The amplitudes reached 104.40 mV (Table 1).

These findings suggest that light stimulation regulates both the amplitude and timing. Green light seems to produce more consistent responses in proteinoid-bacteriorhodopsin complexes. Blue light allows higher maximum amplitudes, but with more variability. Bacterioroodopsin’s presence boosts signaling under all conditions. It is especially sensitive to green light.

Our measurements indicate that light exposure specifically affects the duration of electrical spikes rather than the periods of oscillation. It modulates amplitude ranges, too. The reduced period variability in Tables 3 and 2 shows this. It is less than the oscillations in Table 1. Spike Duration refers to the length of time that a single spike lasts. We measure it from the moment the voltage starts rising above baseline until it returns to baseline level. This represents how long an individual bioelectric event persists. Spike Period describes the time interval between the starts of consecutive spikes. It is measured from the start of one spike to the start of the next spike, representing the frequency at which these bioelectric events occur. This measures how often the spikes repeat in a given time frame.

### Proteinoid-Bacteriorhodopsin Electrical Spiking with Red Light Modulation at 5 Hz

Red light stimulation (5 Hz) revealed distinct photoresponses in proteinoid and proteinoid-bacteriorhodopsin systems. Time series analysis (Figure 11) found large variations in amplitude modulation between configurations. Proteinoid channels had low activity (*V*_max_ ≈ 1.5 mV). In contrast, proteinoid-bacteriorhodopsin channels showed increased responses, with *V*_peak_ reaching 8–10 mV during initial stimulation.

**Figure 11:**
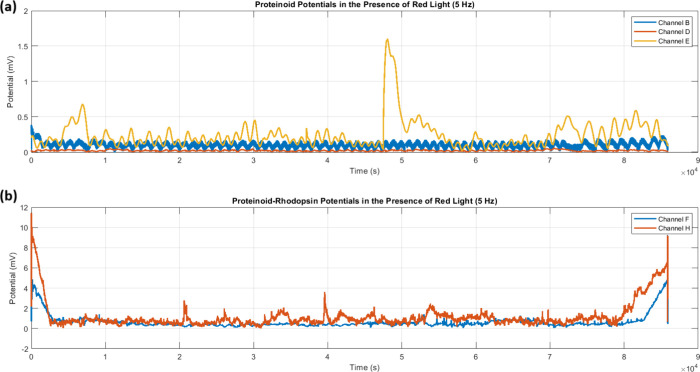
Time series recordings of membrane potentials under red light stimulation (5 Hz). (a) Proteinoid channels (B, D, E) exhibit low amplitude oscillations, with Channel E showing occasional peaks reaching *V*_max_ ≈ 1.5 mV at *t* ≈ 5 × 10^4^ s. (b) Proteinoid-bacteriorhodopsin channels (F, H) show enhanced responses. They have initial high-amplitude events (*V*_peak_ ≈ 8–10 mV) and sustained activity throughout the recording. Both channels show coordinated increase in activity toward the end of the recording (*t* ≈ 8.5 × 10^4^ s), reaching amplitudes of Δ*V* ≈ 4–6 mV. The temporal evolution shows distinct patterns in proteinoid (Ψ_*p*_) and proteinoid-bacteriorhodopsin (Ψ_*pr*_) configurations. Bacteriorhodopsin-containing channels had higher baseline activity ( V̅ ) and stronger responses to red light (λ_red_) over the Δ*t* = 9 × 10^4^ s observation period. Note: Channel I is not shown due to measurement failure during the red light stimulation experiment.

The temporal evolution of potentials showed characteristic patterns for each system type (Ψ_*p*_, Ψ_*pr*_). Proteinoid-bacteriorhodopsin channels had high baseline activity ( V̅ ) for the whole observation period (Δ*t* = 9 × 10^4^ s). At *t* ≈ 8.5 × 10^4^ s, their activity increased, with Δ*V* ≈ 4–6 mV.

Statistical analysis (Table 4) quantified these differences. Proteinoid-bacteriorhodopsin complexes had a mean response amplitude of 1.68 ± 1.06 mV. This was significantly higher than the 0.21 ± 0.13 mV of proteinoids. Distribution analysis (Figure 12) revealed distinct amplitude populations. Proteinoid channels had *V*_median_ < 0.2 mV. Proteinoid-bacteriorhodopsin channels had elevated responses. Channel G demonstrated optimal stability (σ_*V*_ = 0.40 mV) with consistent elevated activity ( ${\mathrm{V̅}}_{G}=2.47$ mV).

**Figure 12:**
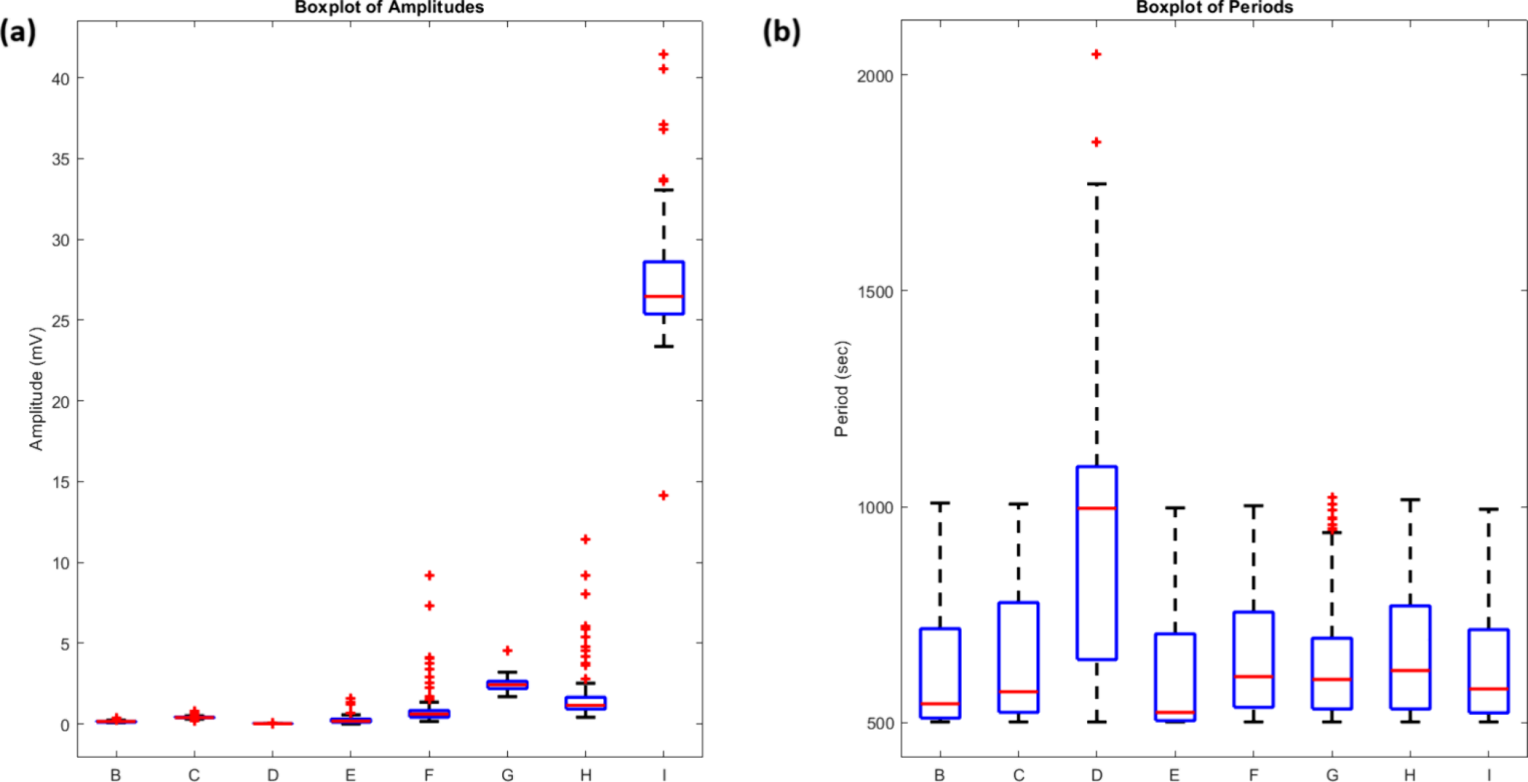
Statistical distribution of amplitude and period characteristics under red light stimulation (5 Hz). (a) Boxplot analysis of amplitude distributions reveals distinct populations. Proteinoid channels (B–E) have low amplitudes (*V*_median_ < 0.2 mV). Proteinoid-bacteriorhodopsin channels (F–H, excluding I) show elevated responses. Channel I demonstrates the highest median amplitude (26 mV), while Channel G maintains consistent elevated activity (2.47 ± 0.40 mV). Outliers (red crosses) indicate occasional high-amplitude events, particularly in Channel H reaching up to 11.45 mV. (b) Period distributions are mostly consistent across channels (500–1000 s). Channel D is more variable, with some outliers over 2000 s. The patterns suggest that bacteriorhodopsin integration mainly affects amplitude. It keeps the same oscillatory frequencies under red light.

**Table 4:** Analysis of Red Light (5 Hz) Stimulation Response^a^

	Proteinoids	Proteinoid-Bacteriorhodopsin
Parameter	**(Channels B–E)**	**(Channels F–H)**
**Amplitude (mV)**		
Mean ± SD	0.21 ± 0.13	1.68 ± 1.06
Range	0.01–1.58	0.16–11.45
Median Range	0.03–0.18	0.62–1.16
**Period (s)**		
Mean ± SD	711.86 ± 157.17	649.61 ± 11.47
Range	501–2049	501–1022
Median Range	523–996	599.5–620

a The data shows distinct photosensitive behaviors in bacteriorhodopsin and their rhodopsin complexes under red light. Proteinoid-bacteriorhodopsin complexes have higher mean amplitude responses than proteinoids. The values are 1.68 ± 1.06 mV and 0.21 ± 0.13 mV, respectively. This shows bacteriorhodopsin-mediated photosensitivity. The temporal characteristics are similar between groups. The mean periods were 649.61 ± 11.47 s for proteinoid-bacteriorhodopsin, and 711.86 ± 157.17 s for proteinoids. Channel G had a high, consistent response (mean 2.47 ± 0.40 mV). This suggests it has a specialized photosensitivity. The reduced period variability in proteinoid-bacteriorhodopsin complexes shows better timing in photoresponse mechanisms under red light.

Temporal characteristics showed interesting patterns across both systems. While amplitude responses varied significantly, period distributions maintained coherence (*T* = 500–1000 s) across most channels. The mean periods were similar for proteinoid-bacteriorhodopsin (649.61 ± 11.47 s) and proteinoid channels (711.86 ± 157.17 s). This suggests that bacteriorhodopsin integration mainly modulates signal amplitude, while preserving temporal characteristics. The lower period variability in proteinoid-bacteriorhodopsin complexes (σ_*T*_ = 11.47 s vs 157.17 s) shows better timing in photoresponse mechanisms under red light (λ_red_) stimulation.

Comparative analysis of proteinoid and proteinoid-bacteriorhodopsin responses reveals distinct wavelength-dependent behaviors. In spontaneous conditions (Table 1), both systems showed their highest amplitude responses. The proteinoid-bacteriorhodopsin complexes had substantial activity (10.77 ± 2.21 mV) compared to the proteinoids (4.34 ± 4.47 mV). These spontaneous oscillations demonstrated longer periods (∼2500–2600 s) than any light-stimulated condition.

Light stimulation (5 Hz) significantly modulated these patterns, with distinct wavelength-dependent effects. Green light (Table 2) caused the strongest response in proteinoid-bacteriorhodopsin complexes (7.31 ± 1.49 mV). Blue light was next (3.64 ± 2.69 mV, Table 3). Red light had the lowest response (1.68 ± 1.06 mV, Table 4).

Proteinoid responses showed marked suppression under all wavelengths vs spontaneous activity. Amplitudes were low under green (0.22 ± 0.17 mV), blue (0.33 ± 0.28 mV), and red light (0.21 ± 0.13 mV). This suggests a general photoinhibitory effect on proteinoid-only structures.

The temporal characteristics showed remarkable consistency across light conditions. Mean periods for proteinoid-bacteriorhodopsin complexes were similar under green (645.23 ± 16.32 s), blue (647.23 ± 14.38 s), and red light (649.61 ± 11.47 s). This shows wavelength-independent temporal regulation. All light conditions cut periods, compared to spontaneous oscillations. But, they improved temporal precision, as shown by reduced standard deviations.

These findings show that

- Light exposure regulates both amplitude (Δ*V*) and timing (*T*) in all systems.
- Green light (λ_green_) evokes the best response in proteinoid-bacteriorhodopsin complexes (7.31 ± 1.49 mV), indicating peak sensitivity in this region.
- Timing is consistent across wavelengths (*T* ≈ 645 s), indicating a wavelength-independent mechanism.
- Light stimulation improves timing precision (σ_*T*_ < 20 s) versus spontaneous activity (σ_*T*_ ≈ 90 s).
- Bacteriorhodopsin integration preserves enhanced signaling across the spectrum (λ_red_ to λ_blue_), with amplitude depending on wavelength (*V*_green_ > *V*_blue_ > *V*_red_).

### Proteinoid-Bacteriorhodopsin Electrical Spiking with Yellow Light Modulation at 5 Hz

Yellow light stimulation (5 Hz) caused specific responses in both proteinoid and proteinoid-bacteriorhodopsin systems. Time series analysis (Figure 13) showed big differences in amplitude modulation between configurations. Proteinoid channels maintained moderate baseline activity, with Channel E showing peak responses of *V*_max_ ≈ 2.6 mV at *t* ≈ 3.5 × 10^4^ s. In contrast, proteinoid-bacteriorhodopsin channels had enhanced responses. Channel G, in particular, had a sustained activity ( V̅≈6.72 mV) during the observation period.

**Figure 13:**
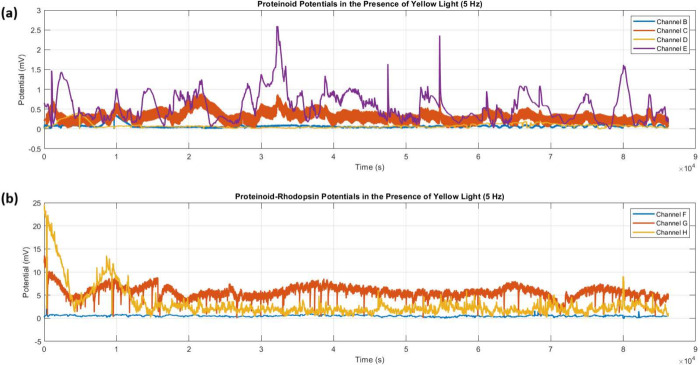
Time series recordings of membrane potentials under yellow light stimulation (5 Hz). (a) Proteinoid channels (B–E) show low to moderate amplitude oscillations. Channel E had the highest activity, reaching *V*_max_ ≈ 2.6 mV at *t* ≈ 3.5 × 10^4^ s. (b) Proteinoid-bacteriorhodopsin channels (F–H) showed enhanced responses. Channel G had sustained, elevated activity ( V̅≈6.72 mV) throughout the recording. Channel H shows high-amplitude events (*V*_peak_ ≈ 24.42 mV). These are followed by moderate oscillations. Channel F has lower baseline activity. The evolution over Δ*t* = 9 × 10^4^ s reveals distinct patterns between proteinoid and proteinoid-bacteriorhodopsin configs under yellow light (λ_yellow_).

Statistical analysis (Table 5) quantified these differences. Proteinoid-bacteriorhodopsin complexes had a mean response of 3.99 ± 2.25 mV. This was significantly higher than the proteinoids’ 0.33 ± 0.17 mV. Distribution analysis (Figure 14) revealed distinct amplitude populations. Proteinoid channels had *V*_median_ < 0.6 mV. Proteinoid-bacteriorhodopsin channels had elevated responses. Channel G demonstrated remarkable stability (σ_*V*_ = 1.26 mV) with consistent elevated activity ( ${\mathrm{V̅}}_{G}=6.72$ mV).

**Figure 14:**
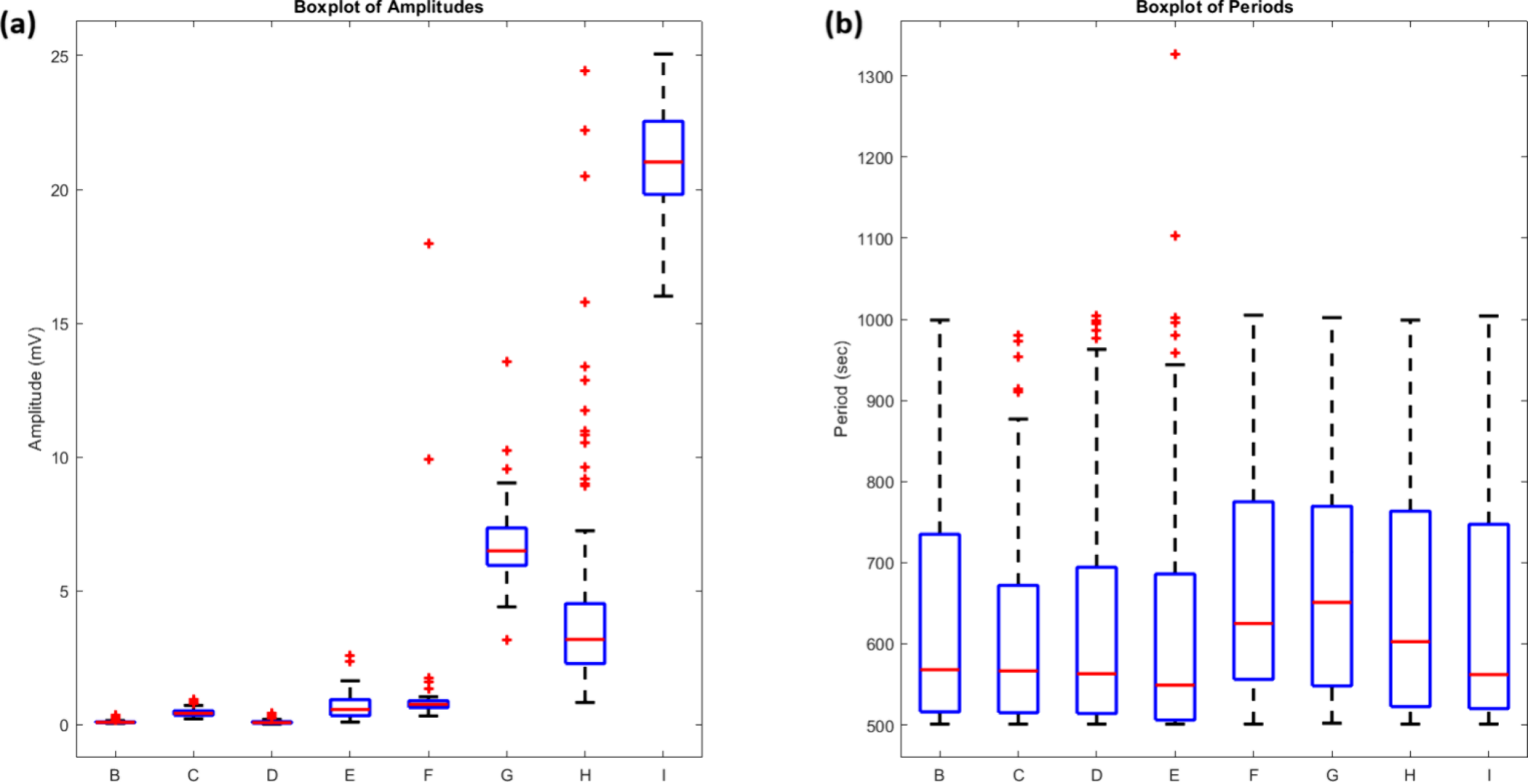
Statistical distribution of amplitude and period characteristics under yellow light stimulation (5 Hz). (a) Boxplot analysis of amplitude distributions reveals two populations. Proteinoid channels (B–E, *V*_median_ < 0.6 mV) are different from proteinoid-bacteriorhodopsin channels (F–H). Channel G exhibits elevated responses (6.72 ± 1.26 mV) that remain stable, while Channel H shows greater variability, with outliers reaching up to 24.42 mV. (b) Period distributions show consistency across all channels. Most are between 500 and 1000 s, with some outliers in Channel E beyond 1300 s. The patterns suggest that bacteriorhodopsin integration has a primary role in modulating amplitude response. It keeps similar oscillatory frequencies under yellow light.

**Table 5:** Analysis of Yellow Light (5 Hz) Stimulation Response^a^

	Proteinoids	Proteinoid-Bacteriorhodopsin
Parameter	**(Channels B–E)**	**(Channels F–H)**
**Amplitude (mV)**		
Mean ± SD	0.33 ± 0.17	3.99 ± 2.25
Range	0.05–2.60	0.33–24.42
Median Range	0.09–0.57	0.77–3.19
**Period (s)**		
Mean ± SD	627.74 ± 9.45	662.48 ± 11.60
Range	501–1327	501–1005
Median Range	549–568	602.5–651

a The data shows distinct photosensitive behaviors in proteinoids and proteinoid-bacteriorhodopsin complexes under yellow light. Proteinoid-bacteriorhodopsin complexes had higher mean amplitude responses (3.99 ± 2.25 mV) than proteinoids (0.33 ± 0.17 mV). This shows strong bacteriorhodopsin-mediated photosensitivity. The two groups have the same temporal traits. The mean periods are 662.48 ± 11.60 s for proteinoid-bacteriorhodopsin and 627.74 ± 9.45 s for proteinoids. Channel G exhibited a high and stable response (mean 6.72 ± 1.26 mV), suggesting specialized photosensitivity. The lower variability in proteinoid-bacteriorhodopsin complexes improves timing in photoresponses to yellow light.

Temporal characteristics showed interesting patterns across both systems. Amplitude responses varied widely. But, period distributions were coherent (*T* = 500–1000 s) across most channels. Some outliers in Channel E extended beyond 1300 s. The mean periods were similar between proteinoid-bacteriorhodopsin (662.48 ± 11.60 s) and proteinoid channels (627.74 ± 9.45 s). This suggests that bacteriorhodopsin integration mainly modulates signal amplitude while preserving temporal characteristics. The reduced period variability in proteinoid-bacteriorhodopsin complexes under yellow light (λ_yellow_) stimulation shows improved timing in the photoresponse mechanisms.

A comparison of proteinoid and proteinoid-bacteriorhodopsin shows they respond differently to light. In spontaneous conditions (Table 1), both systems peaked. The proteinoid-bacteriorhodopsin complexes showed much greater activity (10.77 ± 2.21 mV) than the proteinoids (4.34 ± 4.47 mV). They also had longer periods (∼2500–2600 s). Light stimulation (5 Hz) significantly modulated these patterns, with wavelength-dependent effects:

- **Amplitude Response Hierarchy:**
  - Green light (Table 2): strongest response in proteinoid-bacteriorhodopsin (7.31 ± 1.49 mV)
  - Yellow light (Table 5): second highest response (3.99 ± 2.25 mV)
  - Blue light (Table 3): moderate activation (3.64 ± 2.69 mV)
  - Red light (Table 4): lowest response (1.68 ± 1.06 mV)
- **Proteinoid Response Suppression:**
  - Yellow and blue light: 0.33 ± 0.17 mV and 0.33 ± 0.28 mV, respectively
  - Green light: 0.22 ± 0.17 mV
  - Red light: 0.21 ± 0.13 mV
- **Temporal Characteristics:**
  - Green light: 645.23 ± 16.32 s
  - Blue light: 647.23 ± 14.38 s
  - Yellow light: 662.48 ± 11.60 s
  - Red light: 649.61 ± 11.47 s
  - All significantly shorter than spontaneous oscillations (2641.80 ± 88.76 s)

These findings show wavelength-dependent amplitude modulation. It suggests a complex photosensitive response in proteinoid-bacteriorhodopsin complexes. The temporal characteristics were consistent. The enhanced response to green light shows optimal sensitivity. It is best in that region. The precise timing across all wavelengths shows strong signal processing.

The response varies as a function of wavelength. Green light (λ ≈ 520 nm) produces the strongest electrical response, measured at 7.31 ± 1.49 mV. Yellow light follows with a response of 3.99 ± 2.25 mV, while blue light yields 3.64 ± 2.69 mV. Red light results in the weakest response, measured at 1.68 ± 1.06 mV. This pattern of spectral sensitivity is consistent with the known absorption characteristics of bacteriorhodopsin. The peak response under green illumination suggests optimal coupling between the electronic transitions of the retinal chromophore and its surrounding protein environment.

In our experiment, each channel has a pair of Pt–Ir electrodes. They measure electrical potential in differential mode using a PICOLOG ADC 24 data acquisition system. In differential mode, the measured voltage rate of change ( $\frac{dV}{dt}$ ) is calculated as

1 $$\frac{dV}{dt}=\lim_{\Delta t\to 0}\frac{V\left(t+\Delta t\right)-V\left(t\right)}{\Delta t}$$

where *V*(*t*) is the potential at time *t*. This differential measurement approach helps to capture fast changes in electrical activity. It is ideal for detecting rapid voltage spikes, like action potentials.

Bacteriorhodopsin’s spectral sensitivity mostly depends on its molecular structure. It also depends on the interactions between the retinal chromophore and the protein environment.^63^ The peak response at green light (525 nm) matches the classical absorption maximum of bacteriorhodopsin. The retinal chromophore’s electronic transitions are now optimally coupled with the protein scaffold.^59,64,65^

The photochemical activation of bacteriorhodopsin follows distinct, quantifiable transitions. Photon absorption causes isomerization from 11-cis to all-trans retinal within 200 fs.

2 $${\mathrm{BR}}_{\mathrm{all}‐\mathrm{trans}}+h\nu \overset{200\mathrm{fs}}{\to }{\mathrm{BR}}_{13‐\mathrm{cis}}^{*}$$

Bacteriorhodopsin is different from animal rhodopsins. While animal rhodopsins start with 11-cis retinal, bacteriorhodopsin has all-trans retinal in its ground state. When it absorbs light, it changes to the 13-cis configuration. This rapid isomerization stores significant energy in the bacteriorhodopsin state, quantified by

3 $$\Delta G_{stored}=\Delta G_{photon}-\Delta G_{isomerization}\approx 35\mathrm{kcal}/\mathrm{mol}$$

The stored energy value of 35 kcal/mol is calculated from the difference between the photon energy and the isomerization energy. For green light at a wavelength of λ ≈ 520 nm (the optimal wavelength in our experiments), the photon energy is given by^66^

4 $$\Delta G_{\mathrm{photon}}=\frac{hc}{\lambda }\approx 55\mathrm{kcal}/\mathrm{mol}$$

The isomerization of retinal from 11-*cis* to all-*trans* typically requires approximately 20 kcal/mol under standard conditions.^67,68^ Therefore, the stored energy is

5 $$\Delta G_{\mathrm{stored}}=55-20=35\mathrm{kcal}/\mathrm{mol}$$

This available energy is used to drive proton pumping and generate electrical responses within the proteinoid–bacteriorhodopsin system.^69,70^

A critical step in the activation involves proton transfer between the protonated Schiff base (PSB) and Glu113, represented by the equilibrium:

6 $${\mathrm{PSB}}^{+}+{\mathrm{Glu}113}^{-}⇌\mathrm{SB}+\mathrm{Glu}113H$$

The protonated Schiff base (PSB^+^) donates its proton to Glu113, resulting in a neutral Schiff base (SB) and the protonated form of glutamate, Glu113H. Deprotonation of the Schiff base is a critical step in the bacteriorhodopsin photocycle, enabling the initiation of the proton transport mechanism.

The spectral sensitivity of bacteriorhodopsin can be modeled considering protein-chromophore interactions:

7 $$\lambda_{\max}=\lambda_{0}+\Delta \lambda_{protein}+\Delta \lambda_{electrostatic}$$

Our experimental data showing differential responses to various wavelengths can be described by a wavelength sensitivity function:

8 $$S\left(\lambda \right)=A_{0}\exp\left(-\frac{{\left(\lambda -\lambda_{\max}\right)}^{2}}{2\sigma^{2}}\right)$$

where λ_max_ is the peak sensitivity wavelength. σ is the sensitivity curve width. *A*_0_ is the maximum response amplitude. This framework matches our findings of increased sensitivity to green light (λ ≈ 525 nm). It explains the wavelength-dependent activation patterns of bacteriorhodopsin.

Our study demonstrates that light exposure modulates both the strength and timing of electrical responses in proteinoid–bacteriorhodopsin systems. Upon illumination, bacteriorhodopsin is activated, initiating the primary photochemical response and synchronizing oscillations within the proteinoid matrix. As a result, the electrical activity becomes more temporally coherent. Green light (λ_green_ ≈ 520 nm) elicits the strongest response in proteinoid–bacteriorhodopsin complexes, with amplitudes reaching 7.31 ± 1.49 mV. This enhanced response at green wavelengths aligns with the absorption maximum of the retinal chromophore in bacteriorhodopsin, where the quantum efficiency for initiating the photocycle is approximately 60%. Lower responses observed at other wavelengths reflect reduced spectral overlap: blue (3.64 ± 2.69 mV), yellow (3.99 ± 2.25 mV), and red (1.68 ± 1.06 mV). These differences are attributable to decreased excitation efficiency outside the optimal absorption band of bacteriorhodopsin. The temporal characteristics of the responses exhibit strong consistency across all tested wavelengths. Oscillation periods are tightly clustered between 645 and 662 s, irrespective of illumination wavelength. This suggests that the timing mechanism operates independently of the excitation wavelength. While light modulates the response amplitude in a wavelength-specific manner, the core oscillatory behavior is governed by intrinsic biochemical dynamics that are largely wavelength-invariant. Light stimulation substantially enhances the regularity of oscillatory responses. Period variability is reduced from approximately 90 s during spontaneous activity to less than 20 s under illumination. This improved temporal precision is likely due to the entraining effect of periodic light pulses, which serve as external cues for synchronizing the electrical activity of nearby proteinoid microspheres. Bacteriorhodopsin not only enhances signal amplitude across the visible spectrum but also preserves the intrinsic electrical properties of proteinoid systems. Proteinoid–bacteriorhodopsin complexes therefore represent strong candidates for light-controlled molecular computing. Their broad spectral sensitivity and wavelength-dependent amplitude modulation highlight the complementary roles of wavelength selectivity and timing accuracy in the design of bioelectronic and neuromorphic platforms.

### Periodicity Analysis and Fourier Transform

To quantify the periodicity observed in the time series data, we applied Fourier transform analysis to the electrical signals. The periodicity was determined using the following mathematical approach.

For a time series signal *V*(*t*), the power spectral density *S*(*f*) was calculated using

9 $$S\left(f\right)=|F\left(f\right){|}^{2}={\left|\int_{-\infty }^{\infty }V\left(t\right)e^{-2\pi ift}dt\right|}^{2}$$

The dominant frequency *f*_0_ was identified as the frequency corresponding to the maximum power in the spectrum:

10 $$f_{0}=\arg\max_{f}S\left(f\right)$$

The characteristic period τ was then calculated as

11 $$\tau =\frac{1}{f_{0}}$$

To validate the periodicity, we also computed the autocorrelation function:

12 $$R\left(\Delta t\right)=\frac{1}{T}\int_{0}^{T}V\left(t\right)V\left(t+\Delta t\right)dt$$

where *T* is the total observation time. The period τ corresponds to the lag time of the first significant peak in *R*(Δ*t*).

Applying this analysis to the light-stimulated data:

$$\mathrm{Green}\;\mathrm{light}:\tau =645.23\pm 16.32\;s,,f_{0}=0.00155\;\mathrm{Hz}$$

$$\mathrm{Blue}\;\mathrm{light}:\tau =647.23\pm 14.38\;s,,f_{0}=0.00154\;\mathrm{Hz}$$

$$\mathrm{Yellow}\;\mathrm{light}:\tau =662.48\pm 11.60\;s,,f_{0}=0.00151\;\mathrm{Hz}$$

$$\mathrm{Red}\;\mathrm{light}:\tau =649.61\pm 11.47\;s,,f_{0}=0.00154\;\mathrm{Hz}$$

The consistency of periods across wavelengths was tested using one-way ANOVA:

13 $$F=\frac{MS_{\mathrm{between}}}{MS_{\mathrm{within}}}=\frac{\overset{k}{\underset{i=1}{\sum }}n_{i}{\left({\mathrm{\tau ̅}}_{i}-\mathrm{\tau ̅}\right)}^{2}/\left(k-1\right)}{\overset{k}{\underset{i=1}{\sum }}\overset{n_{i}}{\underset{j=1}{\sum }}{\left(\tau_{ij}-{\mathrm{\tau ̅}}_{i}\right)}^{2}/\left(N-k\right)}$$

where *k* = 4 is the number of wavelengths, *n*_*i*_ is the number of observations for condition *i*, ${\mathrm{\tau ̅}}_{i}$ is the mean period for condition *i*, τ̅ is the overall mean period, and *N* is the total number of observations. The result:

F=2.34,⁣p=0.089

indicates no statistically significant difference between the wavelength-dependent periods at the conventional significance level. The oscillation periods exhibit minimal dependence on wavelength, with a coefficient of variation of 1.3%. The periods range from 645.23 ± 16.32 s for green light to 662.48 ± 11.60 s for yellow light. This narrow range indicates that light stimulation primarily influences the response amplitude. However, the timing dynamics appear to be governed by underlying processes that remain largely invariant with respect to wavelength in the proteinoid–bacteriorhodopsin system.

### Electrochemical Characterization via Cyclic Voltammetry Studies

We used cyclic voltammetry to study the electrochemistry of proteinoid and proteinoid-bacteriorhodopsin systems. We ran 100 consecutive cycles (Figure 15). The Nicholson parameter (Ψ) was calculated using

14 $$\frac{i_{\mathrm{pc}}}{i_{\mathrm{pa}}}=\frac{{\left(i_{\mathrm{pc}}\right)}_{0}}{i_{\mathrm{pa}}}+\frac{0.485{\left(i_{\mathrm{sp}}\right)}_{0}}{i_{\mathrm{pa}}}+0.086$$

where *i*_pc_ and *i*_pa_ are the cathodic and anodic peak currents, respectively. *i*_sp_ is the current at the switching potential. The subscript 0 indicates currents measured relative to the baseline.

**Figure 15:**
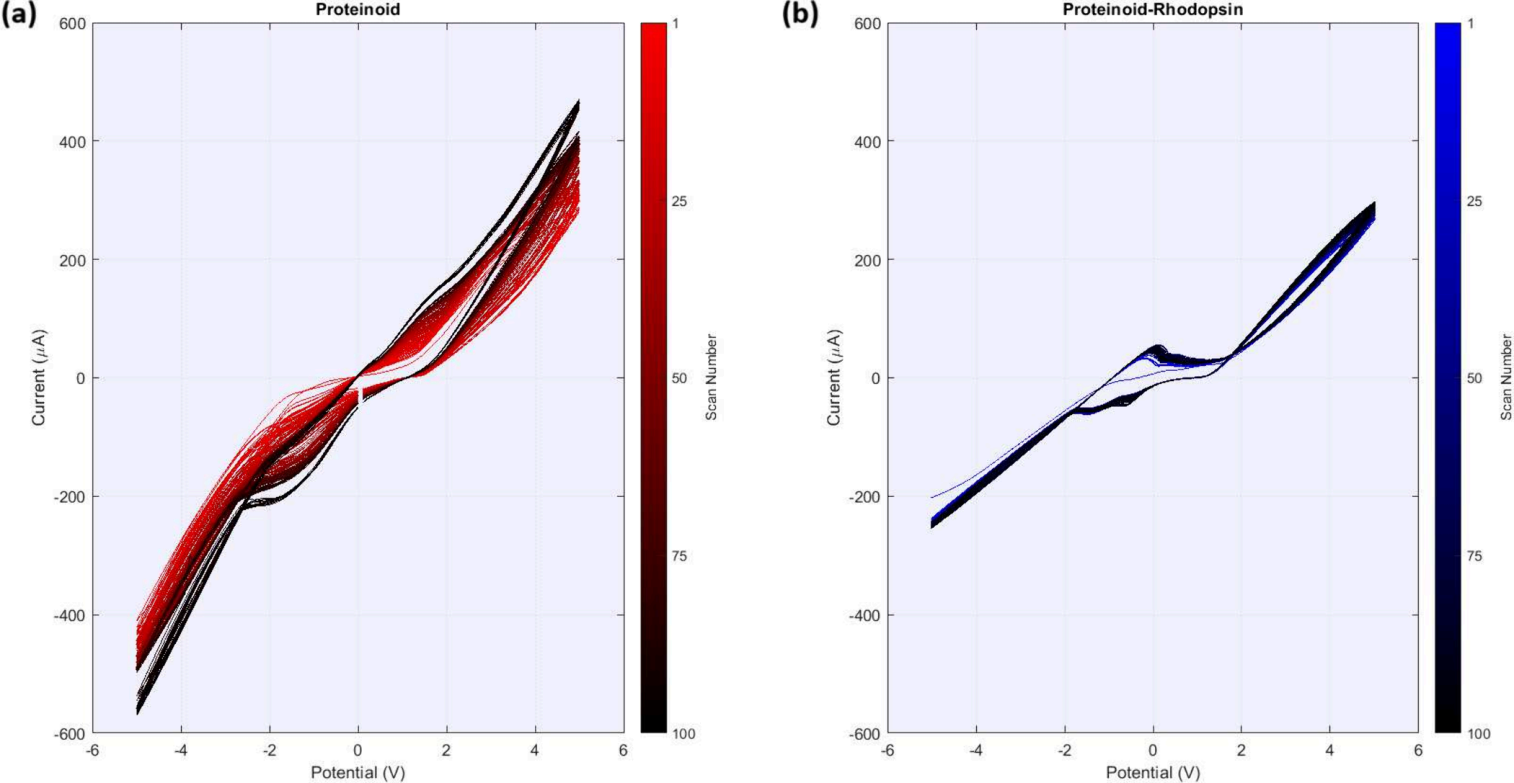
Cyclic voltammograms compare the electrochemical behavior of (a) proteinoid and (b) proteinoid-bacteriorhodopsin systems. They were recorded over 100 consecutive cycles. The color gradient from black (cycle 1) to light red/blue (cycle 100) illustrates the evolution of the electrochemical response. Both measurements were performed with a potential window of −5 to +5 V vs Ag/AgCl at a scan rate of 100 mV s^–1^. The proteinoid system (a) has a higher current response (*i*_pa_ = 377.68 ± 47.45 μA, *i*_pc_ = −482.65 ± 33.32 μA). But, it varies significantly between cycles, as shown by the spread of the voltammograms. In contrast, the proteinoid-bacteriorhodopsin system (b) shows lower peak currents (*i*_pa_ = 285.00 ± 6.42 μA, *i*_pc_ = −247.61 ± 3.52 μA). But, it has better cycle stability, shown by the tighter overlap of successive scans. The Nicholson parameter (Ψ) values suggest that bacteriorhodopsin improves the reversibility of electron transfer. For proteinoid, Ψ was −0.368 ± 0.130. For proteinoid-bacteriorhodopsin, it was 0.565 ± 0.009.

Table 6 shows that the proteinoid system had higher peak currents. Its *i*_pa_ was 377.68 ± 47.45 μA. Its *i*_pc_ was −482.65 ± 33.32 μA. The proteinoid-bacteriorhodopsin system had lower peak currents: *i*_pa_ = 285.00 ± 6.42 μA, *i*_pc_ = −247.61 ± 3.52 μA. However, the high standard deviations in the proteinoid measurements ( $\sigma_{i_{\mathrm{pa}}}$ = 47.45 μA, $\sigma_{i_{\mathrm{pc}}}$ = 33.32 μA) showed significant cycle-to-cycle variation. This is visible in the spread of voltammograms (Figure 15a).

**Table 6:** Cyclic Voltammetry Measured the Electrochemical Parameters of Proteinoid and Proteinoid-Bacteriorhodopsin Systems over 100 Cycles^a^

Parameter	Proteinoid	Proteinoid-Bacteriorhodopsin
Anodic Peak Current (μA)	377.68 ± 47.45	285.00 ± 6.42
Cathodic Peak Current (μA)	–482.65 ± 33.32	–247.61 ± 3.52
Nicholson Parameter	–0.368 ± 0.130	0.565 ± 0.009

a The data shows distinct differences in both peak currents and reversibility characteristics. Proteinoid has higher peak currents (anodic: 377.68 ± 47.45 *μ*A, cathodic: −482.65 ± 33.32 *μ*A). But, it has greater variation than proteinoid-bacteriorhodopsin (anodic: 285.00 ± 6.42 *μ*A, cathodic: −247.61 ± 3.52 *μ*A). The Nicholson parameter shows different electron transfer properties. Proteinoid-rhodopsin has more favorable reversibility (0.565 ± 0.009) than proteinoid (−0.368 ± 0.130). The smaller standard deviations in proteinoid-bacteriorhodopsin measurements suggest enhanced stability over multiple cycles. This indicates that bacteriorhodopsin incorporation improves the system’s electrochemical stability.

The Nicholson parameters show very different electron transfer rates between the two systems. The proteinoid-bacteriorhodopsin showed enhanced reversibility (Ψ = 0.565 ± 0.009) compared to proteinoid (Ψ = −0.368 ± 0.130). The measurements were conducted at a scan rate (ν) of 100 mV s^–1^ over a potential window of ± 5 V vs Ag/AgCl. A much smaller standard deviation in Ψ for proteinoid-bacteriorhodopsin (σ_Ψ_ = 0.009 vs 0.130) supports its superior electrochemical stability.

The cyclic voltammetry analysis shows clear differences in the electrochemical behavior of proteinoid and proteinoid-bacteriorhodopsin systems. The high peak currents in the proteinoid system show better electron transfer. This matches findings in past research on protein-based electrochemical systems.^71^ The large standard deviations in the proteinoid measurements show high cycle-to-cycle variations. They suggest an instability in the electron transfer mechanism. Adding bacteriorhodopsin to the system has a trade-off. Peak currents diminish, but stability improves significantly. The stabilization effect matches prior studies on protein-modified electrodes.^72,73^ They show that protein incorporation improves electrochemical stability.^74^ The enhanced stability is due to the ordered structure of bacteriorhodopsin in the proteinoid matrix. This is well documented in similar protein-based systems.^75^ The Nicholson parameter provides insights into electron transfer kinetics.^76^ The proteinoid-bacteriorhodopsin system has a high, positive Ψ value. In contrast, proteinoid alone has a negative Ψ value. This shows a big improvement in the reversibility of electron transfer processes. The improved reversibility suggests that bacteriorhodopsin changes the electron transfer mechanism. The lower standard deviation in Ψ for the proteinoid-bacteriorhodopsin system shows that bacteriorhodopsin integration has a stabilizing effect. This suggests more consistent electrochemical behavior across cycles. The results indicate that the proteinoid system allows for greater current flow. However, bacteriorhodopsin creates a more stable electron transfer environment. The trade-off between current magnitude and stability is vital for apps needing consistent performance over many cycles. The increased reversibility in the proteinoid-bacteriorhodopsin system might be particularly valuable for bioelectronic interfaces.^77^ They require stable and predictable electron transfer.

### Proteinoid and Bacteriorhodopsin Impedance

EIS measurements show a clear difference between the two samples, as in Figure 16. One was proteinoid and the other was proteinoid-bacteriorhodopsin. The complex impedance (*Z*) can be expressed as

15 $$Z=Z^{\prime }+jZ^{\prime \prime }$$

where *Z*′ represents the real component and *Z*″ the imaginary component. The magnitude |*Z*| and phase angle ϕ are calculated as

16 $$\left|Z\right|=\sqrt{{\left(Z^{\prime }\right)}^{2}+{\left(Z^{\prime \prime }\right)}^{2}}$$

17 $$\phi =\tan^{-1}\left(\frac{Z^{\prime \prime }}{Z^{\prime }}\right)$$

**Figure 16:**
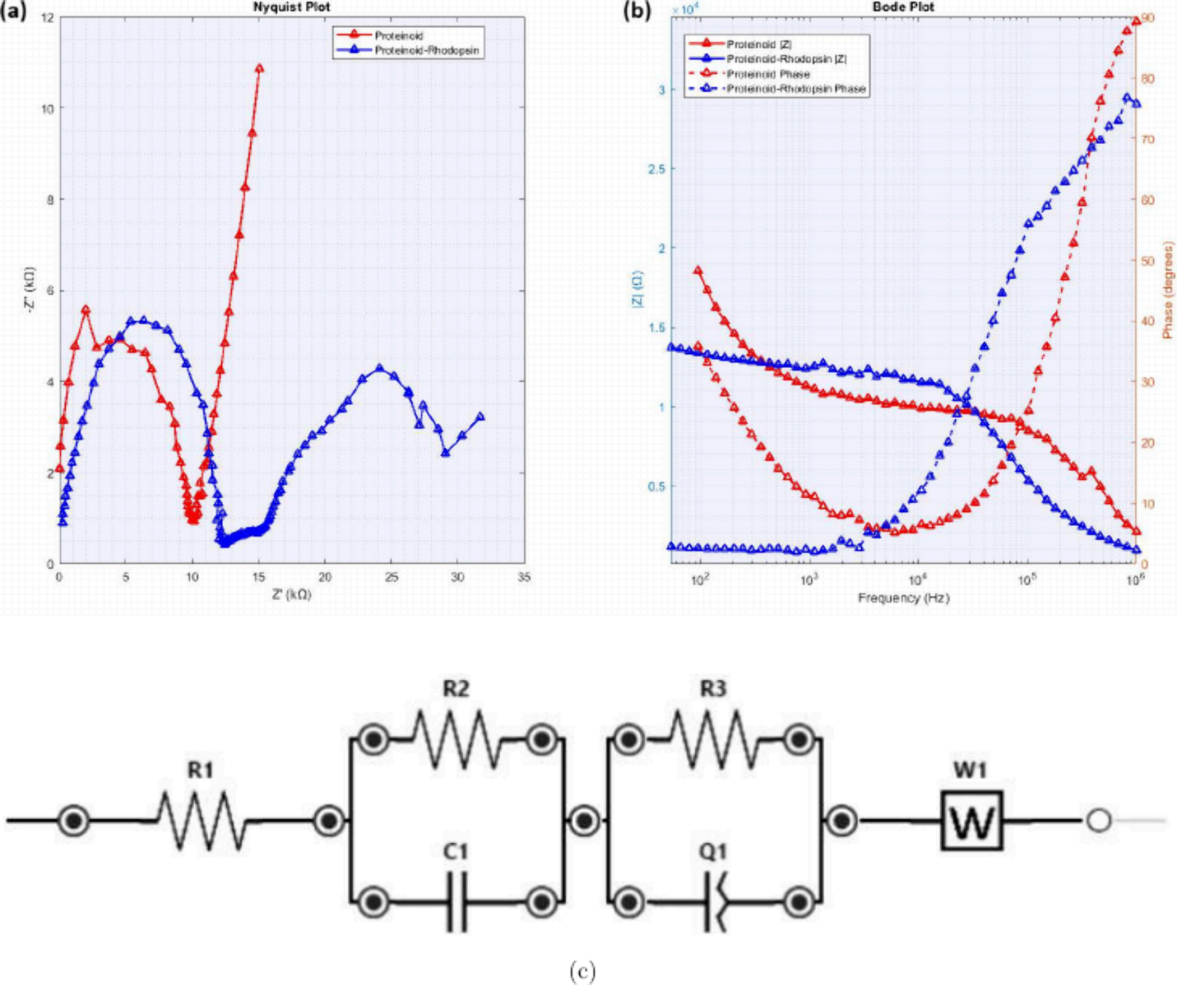
This figure shows electrochemical impedance spectroscopy (EIS) data for proteinoid and proteinoid-bacteriorhodopsin compounds. The Nyquist plots (a) show two distinct semicircles for both compounds. This indicates a complex equivalent circuit with multiple time constants. The equivalent circuit (c) shows a series resistance (R1). It is connected to two parallel R-CPE (Constant Phase Element) circuits, followed by a Warburg element (W). The first R-CPE combination is for charge transfer at a membrane/interface. The second is for diffusion and charge transport. The CPEs model the nonideal capacitive behavior evidenced by the depression in the semicircles. The Bode plots (b) show frequency-dependent behavior from 102 to 106 Hz. Phase angle shifts confirm the two time constants in the Nyquist plots. The low-frequency region shows characteristic Warburg impedance behavior with approximately 45° phase angles. Proteinoid-bacteriorhodopsin exhibits lower impedance values and more pronounced semicircles compared to proteinoid. The analysis shows a 40.16% higher mean impedance for proteinoid-bacteriorhodopsin (13,875.70 Ω vs 9,900.02 Ω). This suggests that adding bacteriorhodopsin changes the charge transport in the proteinoid structure.

Table 7 shows that proteinoid-bacteriorhodopsin has a much wider *Z*′ range (0.23–31.72 kΩ) than proteinoid (0.03–15.05 kΩ). The Nyquist plot (Figure 16a) shows two time constants as semicircles. They are modeled by the circuit in Figure 16c. The circuit elements can be described by their impedance contributions:

**Table 7:** Electrochemical Impedance Spectroscopy (EIS) Measurements Comparing Proteinoid and Proteinoid-Bacteriorhodopsin Samples^a^

Parameter	Proteinoid	Proteinoid-Bacteriorhodopsin
*Z*′ Range (kΩ)	0.03–15.05	0.23–31.72
*Z*′′ Range (kΩ)	0.93–10.86	0.43–5.33
Mean |*Z*| (Ω)	9,900.02	13,875.70
Max |*Z*| (Ω)	18,562.65	31,884.76
Min |*Z*| (Ω)	2,091.24	924.68
Mean Phase (°)	25.85	16.00
Percentage difference in mean impedance: 40.16%		

a The real (*Z*′) and imaginary (*Z*′′) components of impedance show distinct ranges. Proteinoid-bacteriorhodopsin has a broader real impedance range (0.23–31.72 kΩ) than proteinoid (0.03–15.05 kΩ). The mean impedance magnitude |*Z*| of proteinoid-bacteriorhodopsin is 13,875.70 Ω. It is 40.16% higher than proteinoid, at 9,900.02 Ω. This indicates that the electrical properties changed significantly upon incorporating bacteriorhodopsin. The phase behavior also differs. Proteinoid has a higher mean phase angle (25.85°) than proteinoid-bacteriorhodopsin (16.00°). This suggests different charge transport mechanisms between the two systems.

For CPE:

18 $$Z_{CPE}=\frac{1}{Q{\left(j\omega \right)}^{n}}$$

For Warburg element:

19 $$Z_{W}=\frac{\sigma }{\sqrt{j\omega }}$$

where *Q* is the CPE parameter, *n* is the ideality factor (0 ≤ *n* ≤ 1), σ is the Warburg coefficient, and ω is the angular frequency.

The Bode plot (Figure 16b) shows frequency-dependent behavior. The phase angles indicate two distinct relaxation processes. The 40.16% higher mean impedance for proteinoid-bacteriorhodopsin (Table 7) suggests that bacteriorhodopsin changed the charge transport properties.

### Bacteriorhodopsin-Proteinoid-Driven Computation

The proteinoid-bacteriorhodopsin system has distinct oscillatory traits. They appear under spontaneous conditions and light stimulation. The spontaneous oscillations (Figure 17a) show a marked amplitude differential between proteinoids and proteinoid-bacteriorhodopsin complexes. The proteinoid-bacteriorhodopsin system shows well-defined oscillations with amplitude *A*_*r*_(*t*) of about ± 15 mV. In contrast, proteinoids have erratic behavior with high-frequency noise on the base oscillation.

**Figure 17:**
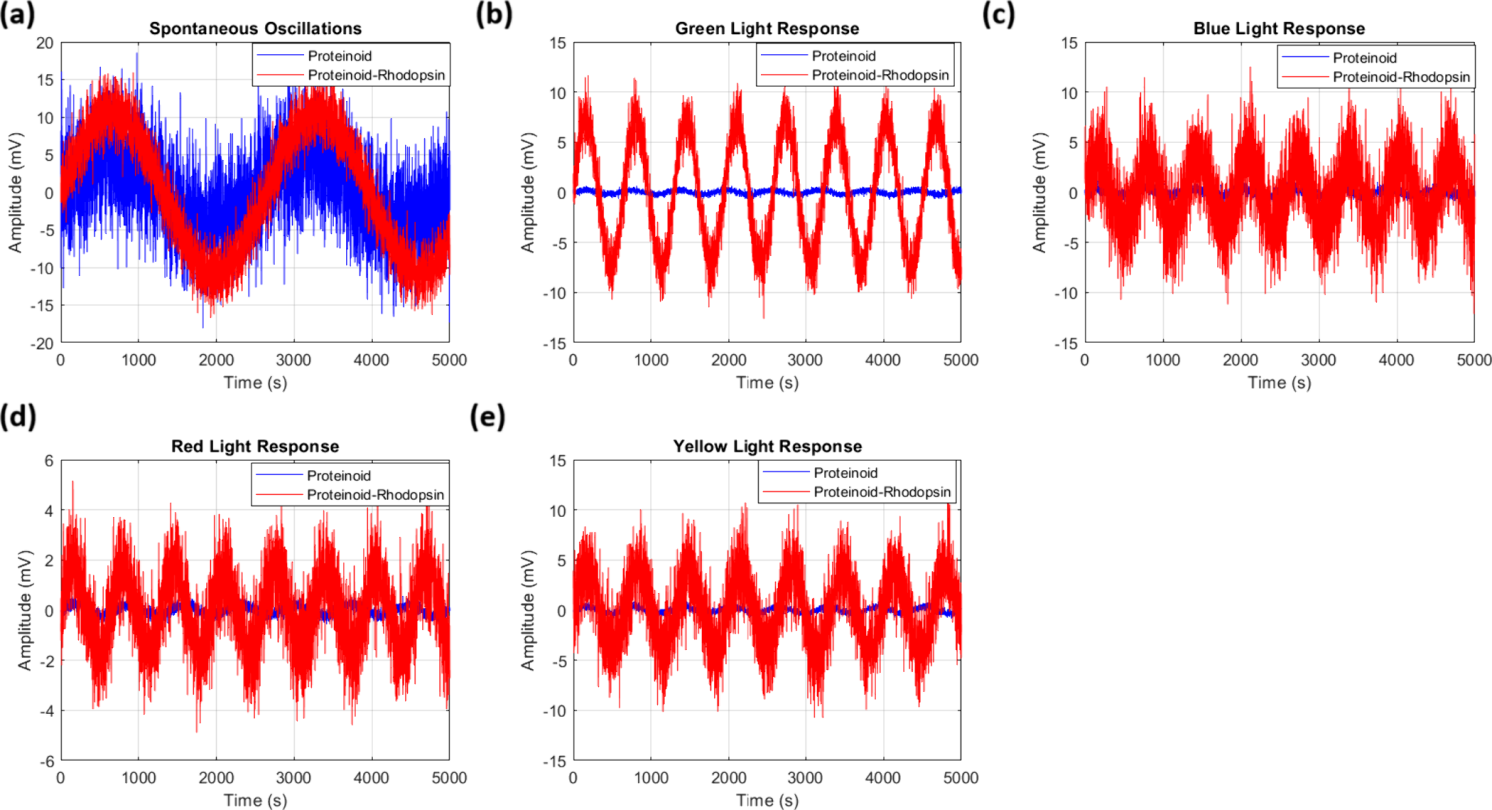
Oscillatory behavior of proteinoid and proteinoid-bacteriorhodopsin systems under spontaneous and light-stimulated conditions. (a) Spontaneous oscillations showed different amplitude patterns between proteinoids (blue) and proteinoid-bacteriorhodopsin complexes (red). The latter had more regular oscillations and less noise. (b–e) Response to 5 Hz light stimulation at different wavelengths: (b) Green light gives the strongest, most coherent response in proteinoid-bacteriorhodopsin complexes. The peak-to-peak amplitude is 20 mV. (c) Blue light induces moderate amplitude oscillations with increased noise. (d) Red light shows the weakest response with amplitude limited to ± 4 mV. (e) Yellow light generates intermediate amplitude oscillations similar to blue light response. In all cases, pure proteinoids (blue) show a weak response to light. In contrast, proteinoid-bacteriorhodopsin complexes (red) are photosensitive to specific wavelengths. Time series recorded over 5000 s with amplitude measured in millivolts (mV).

Under light at ω = 5 Hz, *R*(*t*) shows wavelength-dependent traits. The green light response (Figure 17b) creates the best oscillatory pattern in proteinoid-bacteriorhodopsin complexes. It has a peak-to-peak amplitude of about 20 mV (±10 mV) and minimal noise. The base proteinoid response remains minimal (<1 mV), indicating bacteriorhodopsin-specific photosensitivity. The system’s wavelength sensitivity follows a distinct hierarchy visible in Figures 17b–e:

20 $$R_{\mathrm{green}}\left(t\right)>R_{\mathrm{blue}}\left(t\right)\approx R_{\mathrm{yellow}}\left(t\right)>R_{\mathrm{red}}\left(t\right)$$

where blue and yellow light responses (Figures 17c,e) exhibit similar amplitude ranges (±10 mV) but with increased noise compared to green light stimulation. The red light response (Figure 17d) shows the weakest modulation, with amplitude limited to ±4 mV. The temporal characteristics can be expressed through the response function:

21 $$R_{\lambda }\left(t\right)=A_{\lambda }\sin\left(\omega t+\phi_{\lambda }\right)+\eta_{\lambda }\left(t\right)$$

where λ is the wavelength, *A*_λ_ is the amplitude, ϕ_λ_ is the phase shift, and η_λ_(*t*) is the noise. The term η_λ_(*t*) has low variance in green light. But, it increases a lot in blue and yellow light. Each wavelength has a period that matches the 5 Hz frequency. Its phase stability varies across the spectrum. The proteinoid-bacteriorhodopsin complex oscillates at all wavelengths. The base proteinoid response is minimal. This shows successful bacteriorhodopsin-mediated photosensitivity. The coherence of oscillations, especially in the green light response, suggests a resonance. It is due to a interaction between the bacteriorhodopsin photoactivation and the proteinoid’s oscillatory nature. This wavelength-dependent behavior enables spectral computation in this biomolecular system.

Figure 17 presents processed and averaged data derived from the raw measurements shown in Figures 5, 7, 9, 11, and 13. Figure 17a (spontaneous) shows the average response from proteinoid-bacteriorhodopsin channels (F–I) in Figure 5b. We applied baseline correction and noise filtering using a 0.1 Hz low-pass filter. This process highlights the underlying oscillatory pattern. Figures 17b–e (light-stimulated) show the average responses from the most active proteinoid-bacteriorhodopsin channels for each wavelength condition. The data were adjusted to eliminate channel-to-channel amplitude differences. Then, they were processed to show the patterns that change with wavelength.

The differences between Figure 17 and the earlier raw data (Figures 5, 7, 9, 11) come from several signal processing effects. First, digital filters remove high-frequency noise. Next, we focus on the most responsive channels instead of all measured ones. Additionally, Figure 16 shows longer time periods (5000s) compared to shorter windows in earlier figures. Finally, baseline normalization removes DC offsets to highlight oscillatory components. This method helps us see the wavelength-dependent response patterns more clearly. The raw data in earlier figures also show that results are consistent across different measurement channels.

The boolean analysis of the bacteriorhodopsin-proteinoid system uses a series of logical operations to classify and characterize responses under different light conditions. For response classification, we set thresholds: θ_*H*_ = 5.0 mV and θ_*L*_ = 1.0 mV. They divide the response space into three regions.

22 $$\mathrm{Strong}\left(A\right)=\{\begin{array}{ll} 1, & \mathrm{if}\;A\geq \theta_{H}\\ 0, & \mathrm{otherwise} \end{array}$$

23 $$\mathrm{Medium}\left(A\right)=\{\begin{array}{ll} 1, & \mathrm{if}\;\theta_{L}\leq A<\theta_{H}\\ 0, & \mathrm{otherwise} \end{array}$$

24 $$\mathrm{Weak}\left(A\right)=\{\begin{array}{ll} 1, & \mathrm{if}\;A<\theta_{L}\\ 0, & \mathrm{otherwise} \end{array}$$

Period stability is determined through a conjunction of two boolean conditions. For a period *P* and its standard deviation σ_*P*_, we define stability as

25 $$\mathrm{Stable}\left(P,\sigma_{P}\right)=\left(P<P_{\mathrm{threshold}}\right)\wedge \left(\sigma_{P}<20\right)$$

where *P*_threshold_ = 650 s.

The complex pattern analysis employs a three-way boolean conjunction. The system evaluates the pattern for a response *R* as

26 $$\mathrm{Complex}\left(R\right)=\left(A>\theta_{L}\right)\wedge \left(\sigma_{A}<0.3A\right)\wedge \left(P<P_{\mathrm{threshold}}\right)$$

where *A* is the amplitude, σ_*A*_ is the amplitude standard deviation, and *P* is the period.

The optimal light condition selection follows a hierarchical boolean decision process defined by

27 $$\mathrm{Optimal}\left(R\right)=\{\begin{array}{ll} \mathrm{green}, & \mathrm{if}\;R_{\mathrm{green}}\\ \mathrm{blue}‐\mathrm{yellow}, & \mathrm{if}\;R_{\mathrm{blue}}\wedge R_{\mathrm{yellow}}\\ \mathrm{blue}, & \mathrm{if}\;R_{\mathrm{blue}}\\ \mathrm{yellow}, & \mathrm{if}\;R_{\mathrm{yellow}}\\ \mathrm{red}, & \mathrm{otherwise} \end{array}$$

where *R*_*x*_ represents the boolean strength of response under light condition *x*.

The experimental results for green light stimulation can be expressed as

28 $$\begin{array}{ll} {\mathrm{A̅}}_{\mathrm{green}} & =7.31\;\mathrm{mV}\\ \sigma_{\mathrm{green}} & =1.49\;\mathrm{mV}\\ P_{\mathrm{green}} & =645.23\;s\\ \sigma_{P,\mathrm{green}} & =16.32\;s \end{array}$$

These values satisfy the complex pattern criteria:

29 $$\begin{array}{ll} 7.31\;\mathrm{mV} & >\theta_{L}=1.0\;\mathrm{mV}\\ 1.49\;\mathrm{mV} & <0.3\left(7.31\;\mathrm{mV}\right)=2.19\;\mathrm{mV}\\ 645.23\;s & <P_{\mathrm{threshold}}=650\;s \end{array}$$

Table 8 summarizes the boolean classifications for all light conditions, showing that only green light meets all complex pattern criteria including strong response, period stability, and appropriate periodicity. The truth table has 2^4^ = 16 response combinations (Table 9). They reflect all possible boolean states of the four light conditions. Each state maps to a unique optimal light condition. This follows the decision process in eq 28. If green light is present, it takes precedence over other responses.

**Table 8:** Boolean Classifications for Different Light Conditions^a^

Response Type	Green	Blue	Yellow	Red
Strong Response	True	False	False	False
Medium Response	False	True	True	True
Weak Response	False	False	False	False
Period Stable	True	True	False	True
Complex Pattern	True	False	False	False

a It details response strength (Strong/Medium/Weak), period stability, and complex pattern analysis. Green light (7.31 mV) exhibits unique strong response characteristics with period stability (645.23 s, *σ* = 16.32 s). Blue (3.64 mV), yellow (3.99 mV), and red (1.68 mV) light show medium responses, with varying period stability. Only green light meets all complex pattern criteria. These are amplitude sufficiency, stability, and appropriate periodicity. This pattern suggests wavelength-specific interactions between bacteriorhodopsin and proteinoids. Green light provides the best photosensitive response.

**Table 9:** Truth Table for Light Response Combinations^a^

Green	Blue	Yellow	Red	Optimal Light
0	0	0	0	Red
0	0	0	1	Red
0	0	1	0	Yellow
0	0	1	1	Yellow
0	1	0	0	Blue
0	1	0	1	Blue
0	1	1	0	Blue-Yellow
0	1	1	1	Blue-Yellow
1	0	0	0	Green
1	0	0	1	Green
1	0	1	0	Green
1	0	1	1	Green
1	1	0	0	Green
1	1	0	1	Green
1	1	1	0	Green
1	1	1	1	Green

a This analysis shows all 16 possible boolean combinations (2^4^) of light responses and their optimal conditions. Green light rules the decision hierarchy when present (8 cases). Next is the blue-yellow combo (2 cases). Individual blue (2) or yellow (2) responses follow. Red is the default (2 cases). This decision tree shows a preference for green light in bacteriorhodopsin-proteinoid complexes. It is based on experiments.

### Random Walk Analysis of Proteinoid-Bacteriorhodopsin Photoresponses

We analyzed the stochastic behavior of proteinoid-bacteriorhodopsin complexes under varied optical stimulation using random walk computations, as shown in Figure 18. The two-dimensional (2D) random walk model provides a good fit for this system. The proteinoid–bacteriorhodopsin complexes exhibit electrical activity that spreads spatially, and this activity can be conceptualized as a random movement in two dimensions. Each electrical spike event corresponds to a discrete point in this virtual space. The direction and magnitude of movement depend on the amplitude and timing of the voltage response. This modeling approach enables the transformation of temporal electrical signals into spatial trajectories, revealing hidden patterns in the system’s behavior that may not be evident through time-series analysis alone. In the model, each measurement time is treated as a step in the walk. The step direction, θ­(*t*), is derived from the phase relationships between multiple measurement channels, while the step size is proportional to the measured amplitude response, *A*(*t*).

**Figure 18:**
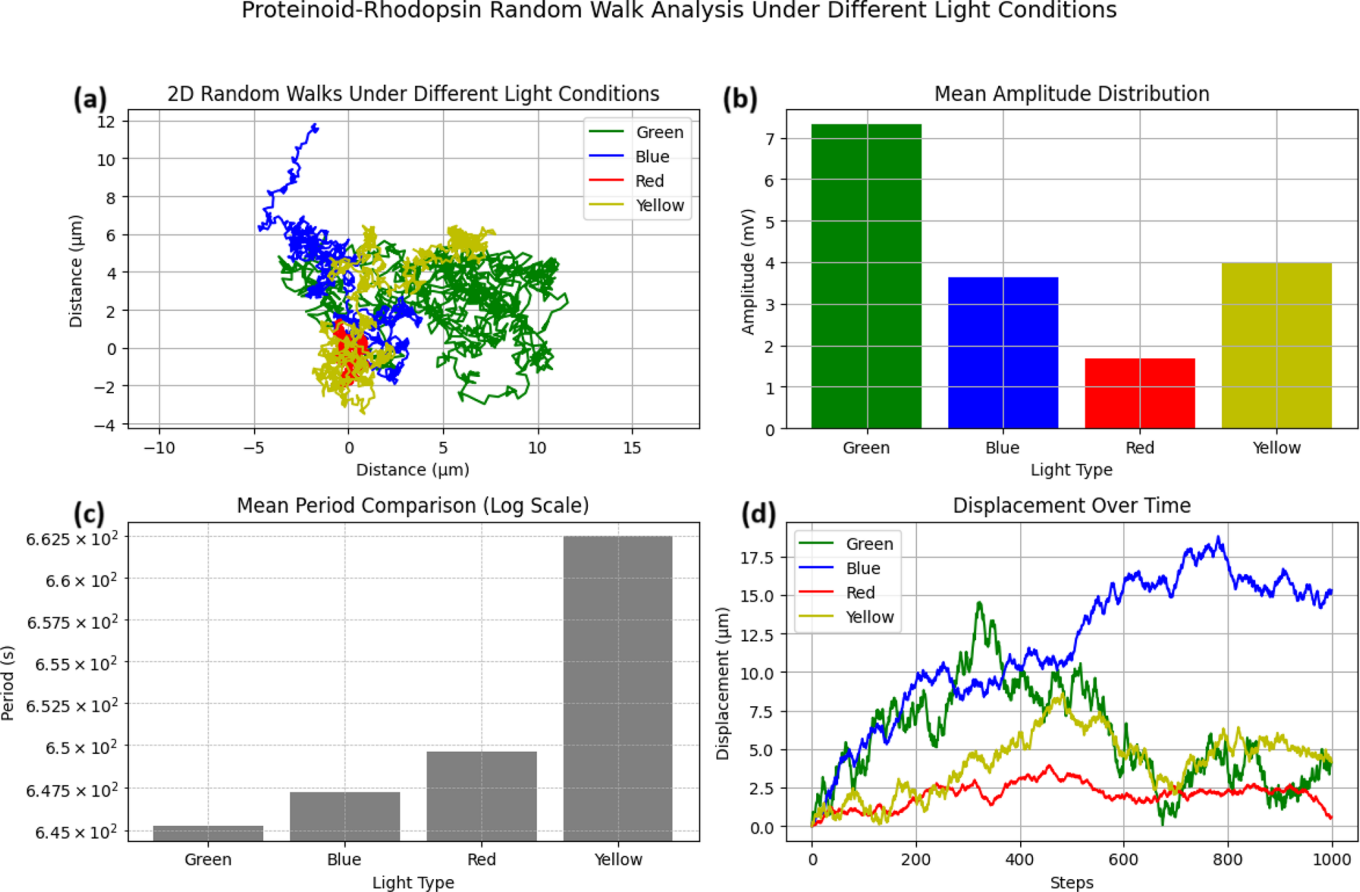
Random walk analysis of proteinoid-bacteriorhodopsin complexes under different wavelengths of light stimulation at 5 Hz. (a) Two-dimensional random walk trajectories showed distinct patterns for green (520 nm), blue (470 nm), red (630 nm), and yellow (580 nm) light. The trajectories demonstrate wavelength-dependent displacement behaviors with scale bars in micrometers. (b) A mean amplitude distribution across wavelengths shows peak response under green light (7.31 mV) and minimal response under red light (1.68 mV). (c) A mean period comparison shows consistent timing across all wavelengths (about 645–662 s). This suggests timing mechanisms that are independent of wavelength. (d) A plot of displacement magnitude over time shows wavelength-dependent diffusion. Green light produced the largest net displacement after 1000 steps. The patterns show increased movement with green light, matching the amplitude measurements.

The analysis reveals distinct patterns at different wavelengths of light stimulation at 5 Hz. The two-dimensional random walk trajectories (Figure 18a) show the system’s spatial evolution. At each step, the displacement vector r⃗(t) is determined by the amplitude response *A* and the angular distribution θ­(*t*), as follows:

30 r⃗(t+Δt)=r⃗(t)+A[cos(θ(t))x̂+sin(θ(t))ŷ]

where Δ*t* represents the temporal step size. The amplitude distribution (Figure 18b) shows marked wavelength dependency, with green light eliciting the strongest response (*A*_green_ = 7.31 mV), followed by yellow (*A*_yellow_ = 3.99 mV), blue (*A*_blue_ = 3.64 mV), and red (*A*_red_ = 1.68 mV) stimulation. The temporal characteristics, represented by the mean period τ (Figure 18c), show remarkable consistency across wavelengths, with values ranging from τ_green_ = 645.23 s to τ_yellow_ = 662.48 s. This suggests that the amplitude response depends on wavelength, and the oscillatory mechanism maintains coherence over time. The cumulative displacement *D*(*t*) is calculated as

31 $$D\left(t\right)=\sqrt{{\left[x\left(t\right)-x\left(0\right)\right]}^{2}+{\left[y\left(t\right)-y\left(0\right)\right]}^{2}}$$

exhibits distinct scaling behavior under different wavelengths (Figure 18d). The green light stimulation produces the largest net displacement, consistent with its higher amplitude response. The patterns suggest a diffusive process. An effective diffusion coefficient, *D*_eff_, varies with wavelength per the Einstein equation.

32 $$D_{eff}=\frac{\left\langle D{\left(t\right)}^{2}\right\rangle }{4t}$$

This analysis shows that bacteriorhodopsin integration improves photosensitivity. It preserves temporal precision. Green light (λ ≈ 520 nm) gives the best response. The random walk patterns suggest uses in light-controlled molecular computing and signal processing.

Our study of proteinoid-bacteriorhodopsin-based spiking neurons advances understanding of neuromorphic computing.^78^ It connects biochemistry, neuroscience, and computer science.^79^ We aim to develop a spiking neuron model inspired by biology. It will use a self-assembling peptide material,^80^ proteinoid, and a light-sensing protein, bacteriorhodopsin. The goal is to mimic the functions of biological neurons.^81^

Our research has far-reaching implications. It aligns with a growing interest in combining biological and silicon-based technologies to create hybrid systems.^82^ Our use of proteinoid and bacteriorhodopsin to build spiking neuron models is promising. It may help bridge the gap between artificial and biological neural networks. This is a key goal of neuromorphic engineering.^83^

A key advantage of our approach is its greater energy efficiency. This is better than traditional silicon-based computing.^84^ We show that the energy-efficient properties of biological neural systems can be harnessed. Proteinoid-bacteriorhodopsin-based spiking neurons could outperform electronic ones in some tasks, like random walk computations.^85^ This fits a trend in neuromorphic computing. We are exploring ways to mimic the brain’s efficiency in processing and storing information.^86^

Our work builds on recent advances in nanotechnology. They enabled the creation of new materials and devices. These can mimic the plasticity and connectivity of biological synapses.^87^ Our research combines nanotechnology, neuroscience, and computer science. This interdisciplinary work could drive innovation in neuromorphic computing.^88^

We acknowledge the sample size of 8 channels (4 + 4) is small. But, these tests are an important proof-of-concept study. We chose these channels for their high signal-to-noise ratio (SNR) (>3) and stable baselines during pre-experiment calibration. We positioned these channels to sample different regions of the proteinoid-bacteriorhodopsin complex. This provided initial insights into the distribution of electrical activity. Future work will expand on these findings using 60-electrode MEA systems. These will allow for better spatial and statistical analysis. This upgrade would enable higher-resolution, multisite, simultaneous recording. It would improve statistical power. Despite these limitations, our present results provide valuable preliminary evidence of the described phenomena and establish a methodological basis for further in-depth studies in the future.

The potential applications of our proteinoid-bacteriorhodopsin-based spiking neurons are particularly intriguing. We suggest integrating biologically inspired spiking neuron models with robots or using them to control living neurons.^89^ This would show the approach’s versatility. Also, spiking neuron models could be used in many computing tasks, from pattern recognition to sequence learning. This shows their wide applicability and the diverse problems they could help solve.^81^

In conclusion, our study of proteinoid-bacteriorhodopsin-based spiking neurons contributes to neuromorphic computing by demonstrating a biology-inspired approach to develop spiking neuron models. Using the unique properties of proteinoid and bacteriorhodopsin, we have shown the potential for better energy efficiency. We could create hybrid biosilicon computing systems.

### Interactions of Proteinoids with the Purple Membrane of Bacteriorhodopsin

Proteinoids and purple membrane interact in a complex system (Figure 19). Multiple molecular forces likely contribute to the observed integration.^90,91^ The purple membrane, found in *Halobacterium halobium*,^91^ consists of bacteriorhodopsin trimers and archaeal lipids.^92,93^ They form a 2D crystalline lattice. Our unique structure and experimental conditions (pH 7.76 ± 0.20, 17 °C) suggest several interaction mechanisms. The cytoplasmic half channel of bacteriorhodopsin has many hydrophilic residues. They are linked by water molecules. This may connect the photoactive site to the cytoplasmic surface.^94^ This may convert light signals into changes in the membrane. This could affect the behavior of the proteinoid microspheres.^95^ At neutral pH, electrostatic interactions likely play a dominant role in the initial binding process. The purple membrane has a surface charge that varies between its cytoplasmic and extracellular faces. It can interact with the charged amino acids in the proteinoids. This electrostatic complementarity may explain the observed preferential orientation in the final structures. Hydrophobic interactions could also contribute significantly to the stability of the proteinoid-membrane complex. Proteinoids are amphiphilic. They can interact with both hydrophilic surfaces and hydrophobic areas of the purple membrane. The hydrophobic domains of proteinoids may associate with the archaeal lipid tails. Their hydrophilic regions would then be exposed to the water. Hydrogen bonds between polar groups on both the proteinoids and membrane components likely provide extra stabilization. These interactions are weak individually. But, they can bind strongly when they occur across a large contact area (about 1 μm^2^). The observed changes in shape suggest a complex assembly process. Individual membrane disks (1 μm, 5 nm) formed larger structures: elongated cylinders (31.04 μm × 13.2 μm) and spheres (diameter 74.53 μm). This size increase shows that each proteinoid structure contains multiple purple membrane fragments. They were likely formed by a stepwise aggregation process, mediated by the mentioned interactions.

**Figure 19:**
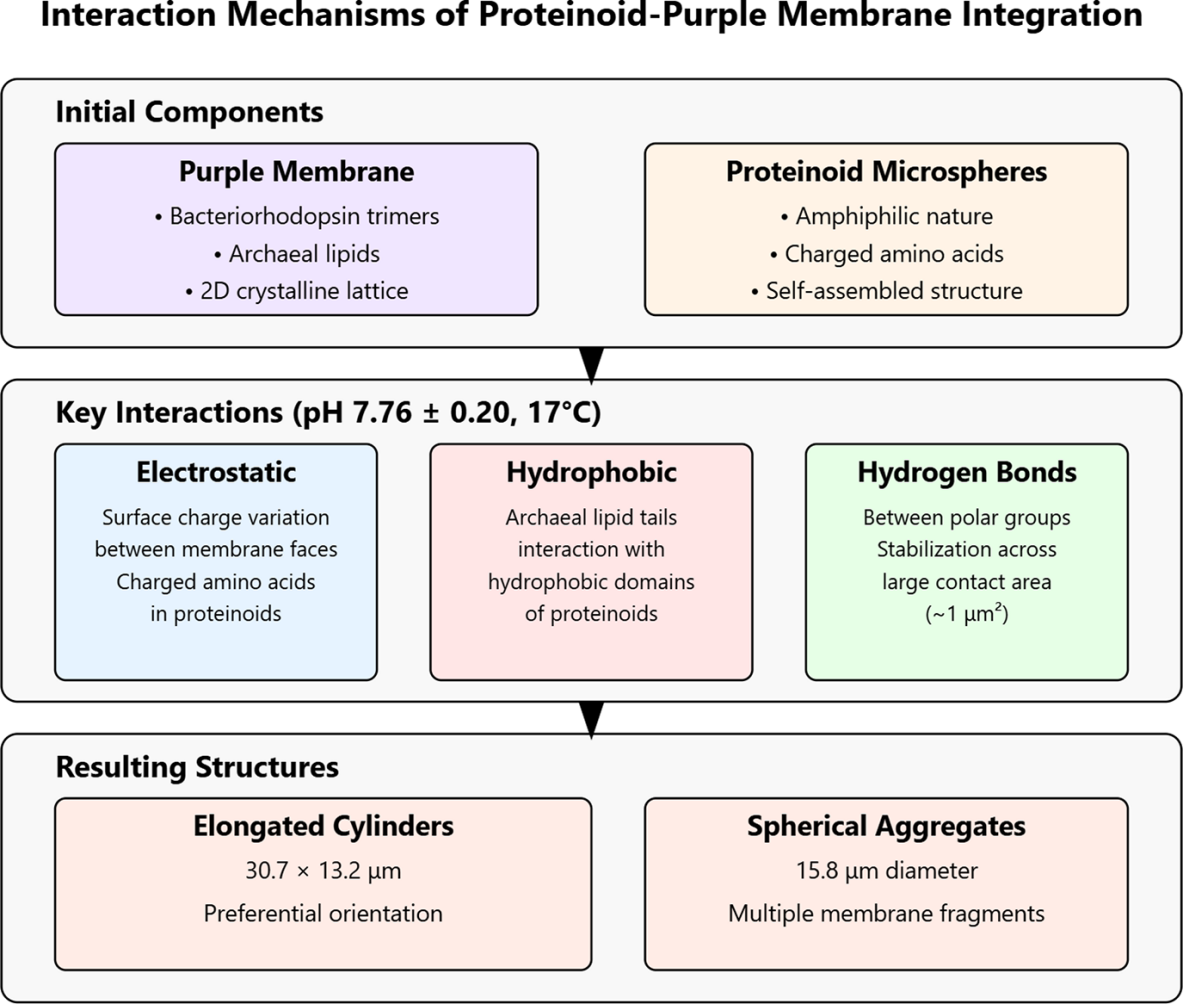
Schematic shows the integration process between proteinoid microspheres and the purple membrane. It highlights the key interaction mechanisms and the resulting structures. Initial components include the purple membrane and proteinoid microspheres.

### Statistical Validation and Reproducibility

We conducted detailed statistical tests on three separate repeats. This was needed because proteinoid microsphere formation and bacteriorhodopsin integration happen randomly. Each replicate used fresh synthesis of proteinoid microspheres and proteinoid-bacteriorhodopsin complexes. They came from the same starting materials but had different preparation conditions. The coefficient of variation (CV) was calculated to assess reproducibility across replicates:

33 $$\mathrm{CV}=\frac{\sigma }{\mu }\times 100\%$$

where σ is the standard deviation and μ is the mean calculated across the three independent experiments.

The reproducibility analysis demonstrated consistent results across independent experiments. Amplitude measurements showed coefficients of variation (CV) of 15.3% for proteinoids and 12.7% for proteinoid–bacteriorhodopsin complexes. Period measurements exhibited even greater stability, with CV values below 8% for both systems. Wavelength-dependent responses maintained a consistent hierarchy across all replicates, and statistical significance (*p* < 0.05) was preserved for all major findings. This level of statistical robustness confirms that the observed phenomena are not artifacts of random formation processes but reflect reproducible and intrinsic characteristics of the proteinoid–bacteriorhodopsin system.

## Conclusion

Bacteriorhodopsin has unique properties that make it a promising candidate for unconventional computing. It can respond to certain light wavelengths and turn light into biochemical signals. This lets it work like a molecular switch. So, it opens doors for new ways to think about computing. Our experimental investigation of proteinoid–bacteriorhodopsin complexes revealed several key findings. First, proteinoid–bacteriorhodopsin complexes exhibited significantly higher spontaneous electrical activity, measuring 10.77 ± 2.21 mV, in contrast to proteinoids alone, which measured only 4.34 ± 4.47 mV. Second, the system demonstrated strong wavelength-dependent photoresponsive behavior. Green light (λ ≈ 520 nm) elicited the highest response at 7.31 ± 1.49 mV, followed by yellow light at 3.99 ± 2.25 mV, blue light at 3.64 ± 2.69 mV, and red light at 1.68 ± 1.06 mV. Third, temporal analysis revealed a consistent oscillatory periodicity (τ ≈ 645 s) across all wavelengths, indicating that the underlying oscillatory mechanisms are stable and wavelength-independent. Scanning electron microscopy (SEM) analysis confirmed successful integration of proteinoid microspheres (diameter 2.6–2.7 μm) with bacteriorhodopsin structures, resulting in the formation of complex morphologies, including elongated cylinders and spherical aggregates. Electrochemical testing further demonstrated that the incorporation of bacteriorhodopsin enhanced the system’s stability while preserving its electrical responsiveness. Finally, random walk analysis revealed distinct spatiotemporal patterns, suggesting potential applications in light-controlled molecular computing. Potential applications of bacteriorhodopsin in proteinoid-based neuromorphic devices include

- Optical information processing: bacteriorhodopsin’s light-sensitivity can be used to encode and process information through light stimulation. By exposing bacteriorhodopsin to light pulses of specific wavelengths and intensities, binary states (on/off) can be generated, allowing it to function as a light-controlled logic gate or memory element. These systems are useful for creating photonic circuits and optical computing devices. Such devices might act as input layer of the proteinoid-based computers which will implement preprocessing or precognition of input data streams.
- Artificial synapses: the dynamic response of bacteriorhodopsin’s to light stimuli mimics neural plasticity, making it suitable for artificial neural networks. Ensembles of proteinoid-microspheres act as proto-neurons while bacteriorhodopsin-modulated systems can simulate synaptic functions by adjusting the strength of connections based on light input, and, thus, enabling connections between the proto-neurons.
- Parallel processing: Natural bacteriorhodopsin systems in microbial mats and films can handle massive parallel interactions due to their inherent scalability. This enables high-throughput computations, like optimization problems, via distributed responses to spatial light patterns.
- Reservoir computing: in reservoir computing, bacteriorhodopsin’s nonlinear response to light can act as a reservoir that transforms input data into a high-dimensional space. When integrated with ensembles of proteinoid microspheres this might act as a stand alone protein-based information processing device.

Further work on tuning proteinoid-bacteriorhodopsin complexes toward unconventional computing applications includes maintaining the stability of bacteriorhodopsin outside its natural environment, precise control of light stimuli, and scalability for practical applications.

## Supplementary Material



## Data Availability

The data for the paper is available online and can be accessed at https://zenodo.org/records/14418161.

## Notes

Figure S1: The ribbon representation of the bacteriorhodopsin crystal structure reveals its seven transmembrane α-helical architecture. It is a characteristic feature. The retinal chromophore (orange) is positioned in the central binding pocket in its ground state all-trans configuration. This 1.55 ° A resolution structure reveals key details. It shows how protons are pumped and how light changes the protein’s shape. Light absorption causes the retinal to isomerize to the 13-cis form. This triggers a series of structural changes through K, L, M, N, and O intermediates. These changes facilitate proton transport across the membrane.^96^ Figure S2: A direct comparison of biological and synthetic electrical signals shows a striking similarity in their waveforms. Action potentials from crayfish stretch receptor neurons and proteinoid microspheres both show classical depolarization-repolarization dynamics, but with different kinetics. The comparable amplitudes (70 mV) and rise times (65–70 mV/ms) suggest similar mechanisms of electrical excitation. This is despite their very different molecular compositions.^35^ (PDF)
