# Supplementary material for: Spiking Neurons Derived from Proteinoid and Bacteriorhodopsin
Source: ACS Appl Bio Mater. 2025 Aug 18;8(9):7953–78. doi: 10.1021/acsabm.5c00964 (PMC12442090; doi:10.1021/acsabm.5c00964)
Supplement: Supplementary file 1 [file mt5c00964_si_001.pdf]

Supporting Information

Spiking Neurons Derived from Proteinoid and Bacteriorhodopsin

Panagiotis Mougkogiannis[1,*] and Andrew Adamatzky[1]

[1]Unconventional Computing Laboratory, University of the West of England, Bristol, UK, BS16 1QY

Email: Panagiotis.Mougkogiannis@uwe.ac.uk

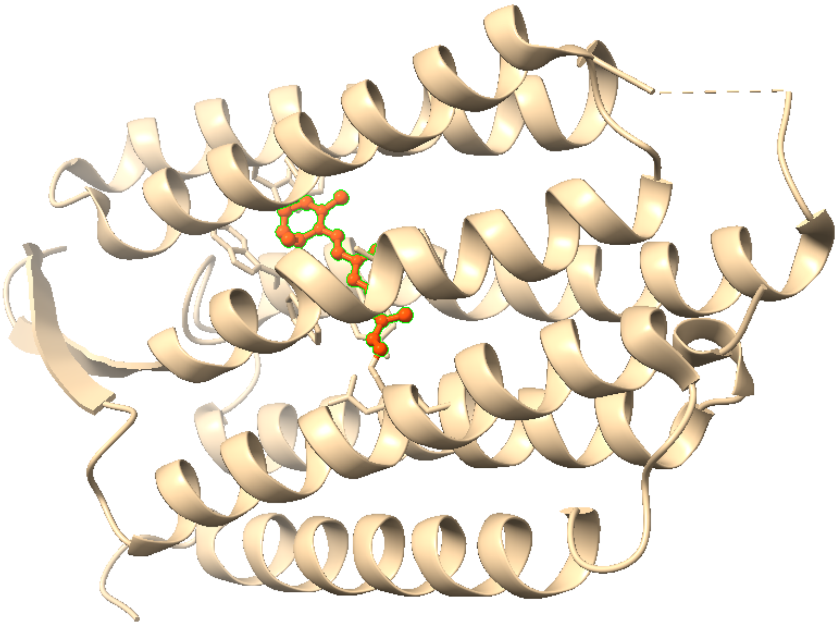

Figure S1: Structural representation of bacteriorhodopsin from *Halobacterium salinarium* (PDB ID: 1C3W). A ribbon diagram shows the monomeric structure with seven transmembrane α-helices (in tan), with the retinal chromophore (orange) prominently displayed in the central binding pocket. While bacteriorhodopsin functions as trimers in the native purple membrane, this figure shows the individual monomeric unit to highlight the structural details of the proton transport mechanism. The retinal molecule is shown in ball-and-stick representation to emphasize its position within the protein scaffold. The structure displays key components required for proton transport, including the retinal chromophore and essential amino acid residues involved in the proton-pumping mechanism. X-ray crystallography at 1.55 Å (R-value = 0.225) reveals the ion transport pathway and lipid–protein interactions within the native membrane. The structure represents bacteriorhodopsin in its ground state (bR_{568}), where the retinal chromophore is in the all-trans configuration. Upon light absorption, the protein undergoes a photocycle involving several intermediates (K, L, M, N, and O states), each associated with specific structural rearrangements and proton transfer events. The depicted conformation precedes the light-induced isomerization of retinal to the 13-cis form, which initiates the proton-pumping cycle.

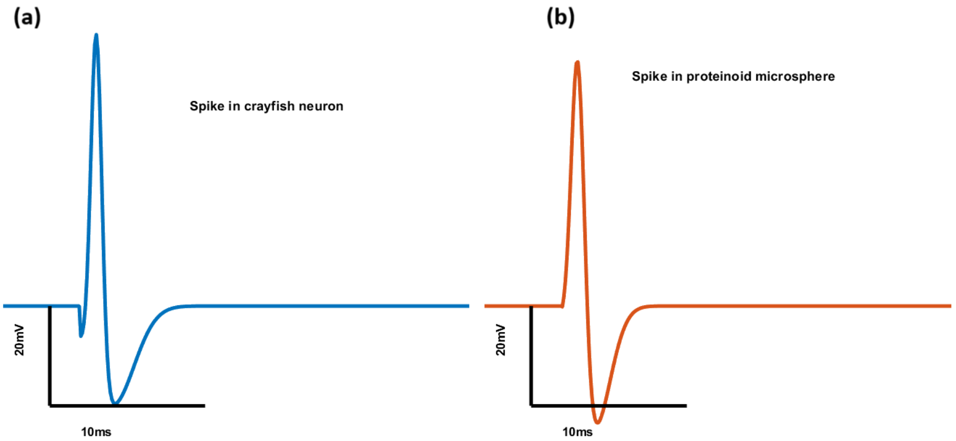

Figure S2: Comparison of electrical signals from a biological neuron and a proteinoid microsphere reveals notable waveform similarities. The traces display action potentials from a crayfish stretch receptor neuron (blue) alongside electrical spikes recorded from a proteinoid microsphere in aqueous suspension (red-orange). Both signals exhibit comparable waveforms, characterized by a rapid depolarization phase followed by repolarization. However, the underlying kinetics differ. In the crayfish neuron, the measurement reflects a transmembrane potential across a lipid bilayer, whereas in the proteinoid system, the signal represents changes in solution potential recorded between electrodes immersed in a colloidal suspension of microspheres. The vertical scale bar is 20 mV, and the horizontal scale bar is 10 ms. Peak amplitudes are similar in both systems, reaching approximately 70 mV. The biological neuron exhibits a slightly steeper depolarization slope ($dV/dt \approx 70$ mV/ms) compared to the proteinoid microsphere ($dV/dt \approx 65$ mV/ms). It is important to note that although the waveforms appear similar, the mechanisms are fundamentally different: the neuronal signal results from ionic currents through voltage-gated channels across a membrane, while the proteinoid signal emerges from collective electrochemical activity within a dispersed colloidal system.[1–4]

References

[1] Fox, S.W., Bahn, P.R., Dose, K., Harada, K., Hsu, L., Ishima, Y., Jungck, J., Kendrick, J., Krampitz, G., Lacey Jr, J.C. and Matsuno, K. (1995). *Experimental retracement of the origins of a protocell: it was also a protoneuron*. Journal of Biological Physics, 20(1), 17–36.

[2] Mougkogiannis, P. and Adamatzky, A. (2023). *Low frequency electrical waves in ensembles of proteinoid microspheres*. Scientific Reports, 13(1), 1992.

[3] Mougkogiannis, P., Ghadafi, E. and Adamatzky, A. (2025). *Bio-inspired cryptography based on proteinoid assemblies*. PLoS One, 20(5), e0324761.

[4] Mougkogiannis, P. and Adamatzky, A. (2025). *Self-Organizing Proteinoid–Actin Networks: Structure and Voltage Dynamics*. ACS Omega, 10(18), 18986–19009.
